# Mathematical model for worm path simulation in 3D

**DOI:** 10.1371/journal.pone.0358065

**Published:** 2026-09-11

**Authors:** Neil Vaughan

**Affiliations:** 1 Department of Clinical and Biomedical Sciences (CBS), University of Exeter, Exeter, United Kingdom; 2 Royal Academy of Engineering, London, United Kingdom; Louisiana State University, UNITED STATES OF AMERICA

## Abstract

This research presents the evaluation of a computer simulation for modelling shapes of paths traversed by various artificial species of worm on a 3D orthogonal grid. These resulting 3D worm trace patterns have not been modelled or analysed before. Simulation software was custom developed in multiple programming languages and platforms, supporting 3D graphics and virtual reality visualisations on various headsets and operating systems. A genetic encoding enables exploration of the full range of 3D worm phenotypes, this research modelled every possible unique worm in simulations up to 53.5 million time-steps. The results show that in this new 3D grid, there are 3239 potentially unique worms. Of the 3239 unique worms, 1882 (58%) terminated at the origin. Also 1352 (41%) of worms entered repeating loops, some complex loops were identified with lengths of up to 296639. Currently 5 worms (0.1%) continue to run chaotically beyond 53.5 million population in simulation runs, and their final outcome still has not been identified yet and remains unknown. Simple rules result in surprisingly complex and intricate movements. Images and unique outlier observations are presented demonstrating complex 3D patterns that emerge.

## Introduction

WORMS produce trails or paths behind them when they move. The worm path patterns have been previously studied [1] including fossilised worm paths of extinct species [2]. Individual species of worm can be identified from their unique path patterns [3]. The nutrients that worms feed on are limited, and are distributed unevenly, usually with some patches of higher concentration. Therefore, rather than moving in straight lines, worm paths benefit from meandering behaviours [3] which increase the chances of both finding a high density nutrient patch, and staying within it for a longer time to ensure that the maximum nutrition is gained. This is also achieved by avoiding any re-tracing of paths that have already been traversed, because no nutrient will remain there. This rule is strictly enforced in computer simulations so that a worm is not allowed to retrace any path that it has previously traversed. Previous worm simulation research [4] has used computer modelling or even physical robots [5] to reproduce worm path patterns. Typically computer models have focussed on triangular [6], hexagonal grids [7] which have 6 paths per step, producing around 400 unique worms [8]. In this research, similarly 6 paths are available per step, however this proposed novel 3D model instead uses a cubic orthogonal grid which enables worm paths to be modelled in 3D for the first time, rather than being restricted to planar motion as in previous hexagonal models and this increases the number of unique worms to 3239. This article is an extension to our previous research on 2D worms which used a novel 8 path grid [9] which has source code available [10]. The source code of our current 3D worm implementation has also been made available [11], using JavaScript, C and Java, supporting Windows and Android with virtual reality (VR) 3D graphics.

### Introduction to 3D model

**Fig 1:**
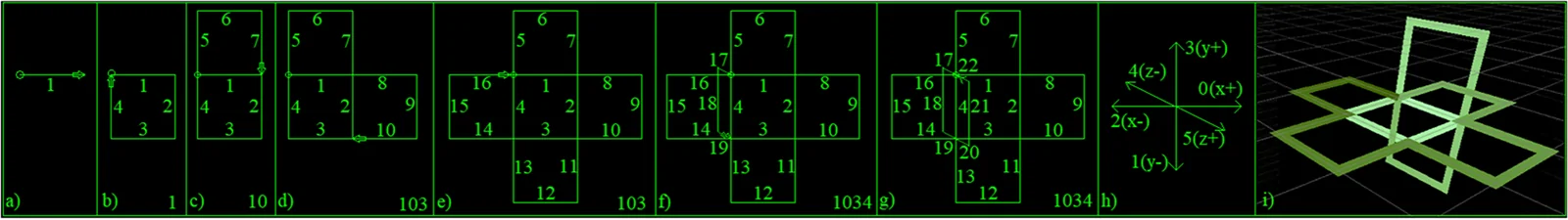
An illustration showing how worms are built: **(a-g)** The worm building process using the proposed algorithm step-by-step, with worm 1034 as an example. The circle indicates the origin (0,0), the arrow shows the current position and direction of the worm. The numbers show the order in which the lines build up from 1 to 22. (h) The 3D axes labelled as used in the implementation. (i) the completed worm path rendered in VR.

This research developed a new 3D model for worm path simulation. The details of the axes are shown in Fig 1h. Paths are numbered from 0 to 5, such that 6 paths join at each grid-point. This 3D format follows the main conventions introduced in previous 2D worm triangular grids [7,8]. The numbering of paths is dependent upon and relative to the present direction and rotation of the worm, such that path 0 will always be straight ahead and path 2 will always be unavailable due to having been traversed behind the worm on the previous move. The four alternative paths: 1, 3, 4 and 5 all represent 90 degree turns in the various directions: up, down, left and right (Fig 1h). When a decision is made about which direction to turn, this is called a rule. Each new rule is saved and that worm must then in future apply the same rule whenever the same set of available paths is encountered. For example, this means that if only paths 4 or 5 are available and the worm chooses path 4 (Fig 1f), then it must in future always choose path 4 if again only paths 4 or 5 are available. The worm continues in this way and if a situation is encountered where no paths are available, the worm terminates [12]. The process of growing 3D worm paths is illustrated in Fig 1. In (a), one line is drawn along the positive x axis, this always occurs to initialise all worms. (b), the first rule 1 is created which results in three 90 degree turns around z axis, drawing a square and arriving back at the origin. (c) A new rule 0 is created which results in line 5 along positive y axis. Rule 1 then creates lines 6 and 7 to re-join the initial square, having created a ‘figure of eight’ pattern. (d) A new rule 3 results in a turn for line 8 along positive x axis, followed by lines 9 and 10 due to rule 1. In (e), rules 3 and 1 reoccur twice more, returning to the origin. In (f), a new rule 4 creates line 17 along negative z axis, followed by rule 1 creating lines 18 and 19 to re-join the square. At this point, path 0 is the only path available so it is chosen automatically without setting a new rule. In (g), rule 1 re-occurs twice, returning to the origin. As no paths are available, the worm 1034 terminates T at the origin with population 22. Population means the number of lines in the worm. Fig 1i shows the worm rendered in the VR software. This is a common worm pattern produced in various rotations and symmetries from several 4 rule worms including: 1034, 1035, 3015 and 4053.

### Categories for termination

Each worm that is run, can be classed into one of five categories (T, L, R, N, I) according to the worm outcome. In practice, with recursive runs, all category I and N worms are unnecessary. Category T: terminated. This occurs when a worm gets stuck with no paths available. Termination can only ever occur when the worm has returned back to the origin (0,0,0) because only then the number of available paths is an even number. Category L: looping. The worm enters a repetitive loop such as drawing repeating zigzag or growing square patterns. These are usually automatically detected by the developed loop detection algorithm. Category R: running. The worm still continues to run and has to be stopped at some population limit, in this case 12 million, due to a lack of time or computing power [13]. The true outcome of these worms is unknown and is still to be determined in future research [14]. Category N: needed more rules. This indicates an incomplete worm, a parent worm, or a branch node. The children worms of category N worms do subsequently need to run to identify the leaf nodes and determine their categories. Category I: invalid rule. This occurs if a rule is set in advance, which is not possible. For example worm 11 is invalid, because after path 1 is traversed the first time, path 1 would not be available a second time. This category of worm is automatically detected within the custom algorithm.

### Rotations and symmetries

Each worm has a number of rotations and symmetries which will produce the same worm path pattern with a different worm code. This also occurs on 2D grids [7]. For example, mirror images can be created by swapping all rules 5 and 3, and also swapping rules 4 and 1. Worm 1034 (Fig 1i) is a mirror image of worm 1035. The mirrored and rotated versions of worms are occasionally useful for observing worms from different directions which can help understand their shapes and properties. All worms starting with rule 1, are duplicated in mirror image worms starting with 3, 4 and 5. The main branches that are mirror images, within worms starting with rule 1, include: 15 and 14, 105 and 104, 135 and 134, 1035 and 1034. These known duplicates were filtered out, which resulted in the list of 3239 unique worms, but some duplicates may remain at deeper levels.

### Theoretical maximum rule depth and number of worms

It can be shown that no worms in this 3D grid can use more than 13 rules. Worms have 6 binary sensors but path 2 is always unavailable, which leaves 2^5^ possible rule choices. In theory up to 32 binary rules are available, because there are maximum of 32 possible binary rule representations from 00000 to 11111. However, half of these rules have an even number of paths available, so only 2 of these can be used per worm, because an even number of available paths can only occur when passing through the origin, which cannot happen more than twice. Of the remaining 16 rules with an odd number of paths, 5 of those rules have only 1 path which are automatically selected without rule setting. One rule has 5 paths which is the first rule, it is restricted to 1 and it does count as a rule. Additionally, this leaves 10 rules with 3 paths. In addition a worm could use 2 rules with an even number of paths: 4 and 2 which can occur. This shows that the total maximum number of rules that could be used by one worm is restricted to 13. In practice, no more than 11 rules have been observed in one worm. Possibly some of the 5 worms currently remaining in category R may require a 12th or 13th rule if run for a longer time, but this seems unlikely since most rules are required within the first few thousand steps after starting.

## Materials and methods

All simulations and source code in 3 languages were developed and run on a HP Windows 10 laptop 64 bit with an Intel Core i7-8664U CPU @ 1.9GHz and 32GB RAM. This CPU supports a maximum turbo frequency up to 4.7GHz, which may contribute towards increased speeds when running multiple worms simultaneously. The worm simulation code was custom developed for this project and ported into several languages, including ANSI C, C# for Unity and android, and JavaScript for web browsers. The JavaScript version [11] can run on any HTML web browser, so the 3D worms can be accessed and viewed from any smartphone, tablet, laptop or any device with a browser, including iPhone Apple Safari. A full set of all 3D worm images has also been made available online [15]. The C# version runs as VR on windows 64-bit as a compiled .EXE file and was also ported into an android app as an .APK file (available online) [11] which can be installed in the android operating system (OS) on most smartphones or tablets. The C# code has virtual reality (VR) support. This gives 3D VR depth perception so a user can be immersed within the mathematical worm structures, and this was tested using several VR headsets including google cardboard, Oculus Rift DK2, Oculus Quest, Oculus Go, Samsung Gear, HTC Vive and HP Windows mixed reality. Videos showing graphics from the VR app are available online. The 3D version is also compatible to view without a VR headset. For viewing 3D structures on a 3D monitor, a 3D to 2D conversion method was custom developed to produce a rapid 3D perspective within a 2D graphics environment using a perspective projection plane, when displayed on a 2D computer or smartphone screen. This effect is produced by restricting the width and height (x and y) of lines that are drawn to be inversely proportional to the distance (z) from projection plane. For testing the code, some known worm patterns were run in all versions of the code. This confirmed that all code versions reproducibly recreated the same worm pattern consistently across all platforms and operating systems. Some variations in the perspective of visualisations between platforms were noticeable and unavoidable due to inherent variation in graphics rending methods in the various supported operating systems and platforms. The initial worm algorithm ran more slowly when simulating large worms. Therefore, in the current updated C version, a custom bubble sorting algorithm was developed to store and retrieve the traversed paths within a list array. This works magnitudes faster by ordering the smallest x coordinate in each x, y, z pair. This enables faster speeds when checking if a path has been previously taken. This uses c function memmove() to quickly shift the whole contents of the array memory block, starting from the char after the position when inserting a new path. Two different worm searching methods were used: random and recursive. Random means picking a rule at run-time when a rule is required as the available paths are known. Random always results in a valid worm being produced so there were no category I worms, but often by chance it repeats the same worm more than once. Alternatively, recursive search means running every worm starting from 1 and checking all children of each worm iteratively, so that every possible worm code is run once and only once, in order from start to finish. Recursive is only feasible if the search space is small enough to run every worm within time frame. In this 3D worm space, recursive search was feasible, so it was completed. As this model has 6 paths to choose per step, it has smaller dimensionality than other grids with larger number of paths. Therefore it was possible to run a full recursive search of every 3D worm. The original Paterson’s worm model with 6 path triangular 2D grid had around 400 unique worms. This 3D grid has more unique worms, around 3239, although some may be duplicates. Identification and removal of mirror image worms was completed, reducing the number of possible unique worms to be modelled, as detailed in later results section, but some duplicate worms could remain.

### Loop definition and automatic detection

Worms that end in category L are defined as having entered infinite loops. The definition of a loop is that the order in which rules are activated consists of a repetitive cycle. An algorithm for automatic loop detection was developed. The purpose of loop detection is that long running loops can be interrupted soon after a loop begins, to reduce unnecessary computing time and worms can then be classified as L algorithmically rather than manually. The loop detection method works by storing every rule activation that occurs during a worm run, in an ordered array. Then a search is performed backwards from the most recent rule activations, to identify any repeating sequences in the array. A sequence length must be greater than 2 rules and has to repeat at least 4 times to be qualified as a loop. When a loop is found, the length is recorded in a log file and the worm run is stopped and labelled as category L. However, on large worms this loop detection algorithm became the substantially slowest part of the simulation, so loop searches were performed at less frequent intervals, which means that some loops are missed and those worms have to be manually labelled as loops afterwards.

### Pruning methods for worms of known outcome

Pruning methods were developed to reduce the total number of worms required to run. This includes by identifying that it was unnecessary to run children of worms in category T, L and R because their result can already be inferred from the known category of their parent worm. Therefore, only worms ending in category N need to be recursively run to include their children. Also worms remaining in category R could in future be run again to a larger population with higher computing power or algorithm such as hashlife [14].

## Results

### Looping worms – category L

**Fig 2:**
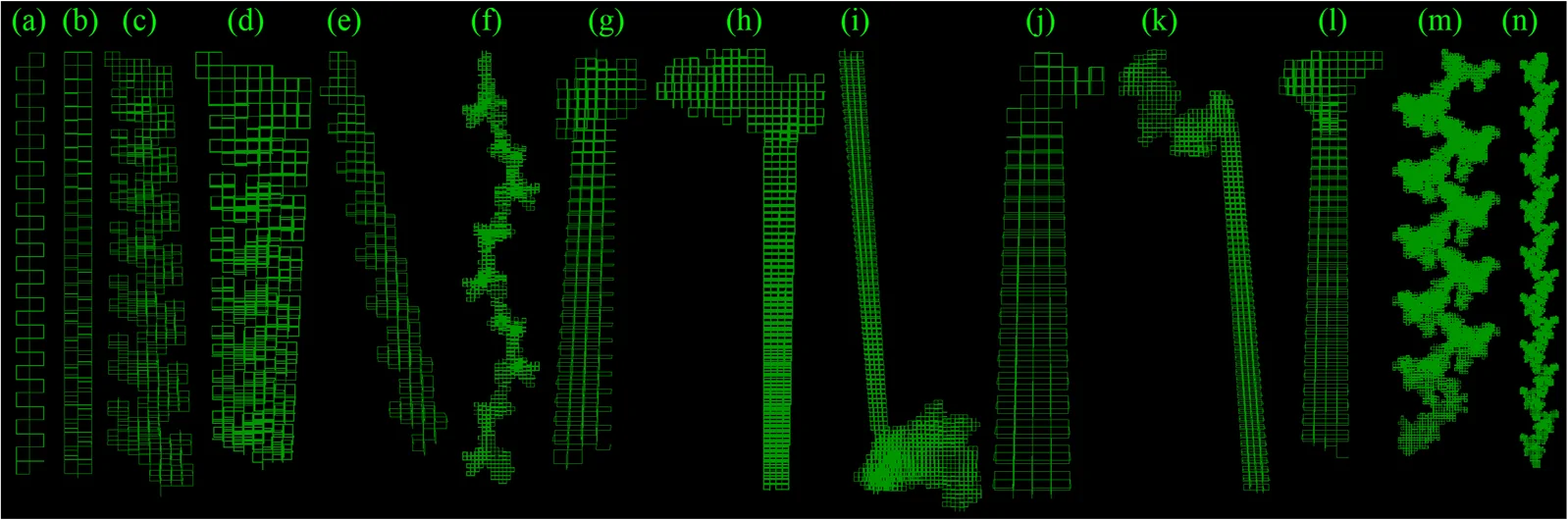
Various worms that create looping path patterns: (a) 104033 at population 220 with loop length 12, (b) 104045 at population 220, loop size 12, (c-d) 134305303 at population 2500 with loop length 132, (e) 135304411 at population 1000 and loop length 99, (f) 10403044545 at population 7200 and loop length 641, (g) 10403535513 at population 1500 and loop length 30, (h) 10403535541 at population 4500 and loop length 17, (i) 10404343501 at population 15000 and loop length 17, (j) 10404343543 at population 1000 and loop length 17, (k) 10404343545 at population 6500 and loop length 17, (l) 14545355445 with population 2300 and loop length 17, (m) vertical spiral type 10403044351 with population 50,000 and loop length 3866, (n) vertical worm 10403534500 with population 50,000 and loop length 1223.

**Fig 3:**
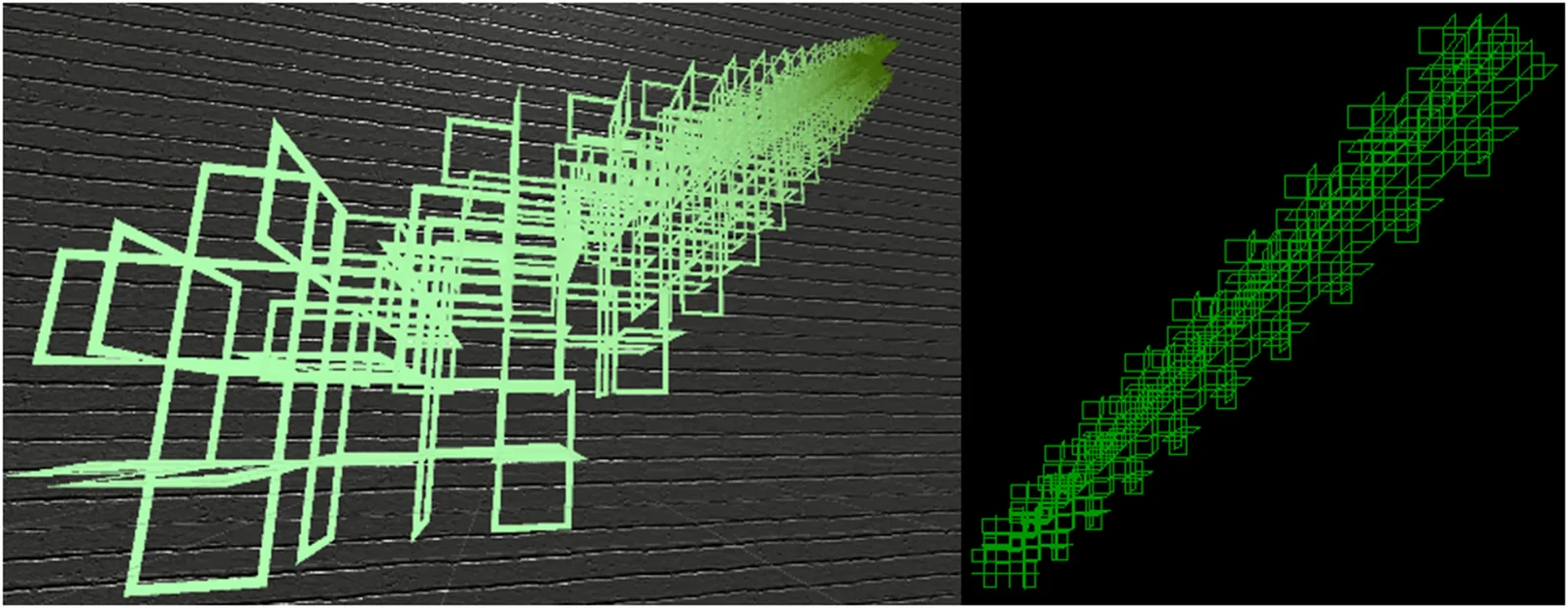
Looping worm: (a) Worm 1454431, in category L, with 7 rules produces an infinite loop of size 40, rendered in virtual reality software. (b) The same worm rendered in C code.

An example of a worm the creates a looping path is a ladder builder worm 105054 (Fig 2a) which expands infinitely in one direction. Previous research on simulating worms in a triangular grid [16] and square diagonal grid [9] also found similar looping worm patterns in 2D, but these are the first found in 3D. Repetitive loops occur with various lengths and complexities (Fig 2b-g). Somewhat longer and more complex loops exhibit intricate structured and spiral patterns (Figs 2f, m and n). Some looping paths are vertical, others horizontal or on the z axis, while some loops occur diagonally (Fig 3), and some are at intermediate angles (Fig 2e). Looping worm patterns were also rendered with VR software in C# (Fig 3a), confirming that the patterns it produces are of identical structure to those produced within C software (Fig 3b). This type of one directional looping is the most common type of loop and makes up around 99% of the looping worms.

### Square building loops

**Fig 4:**
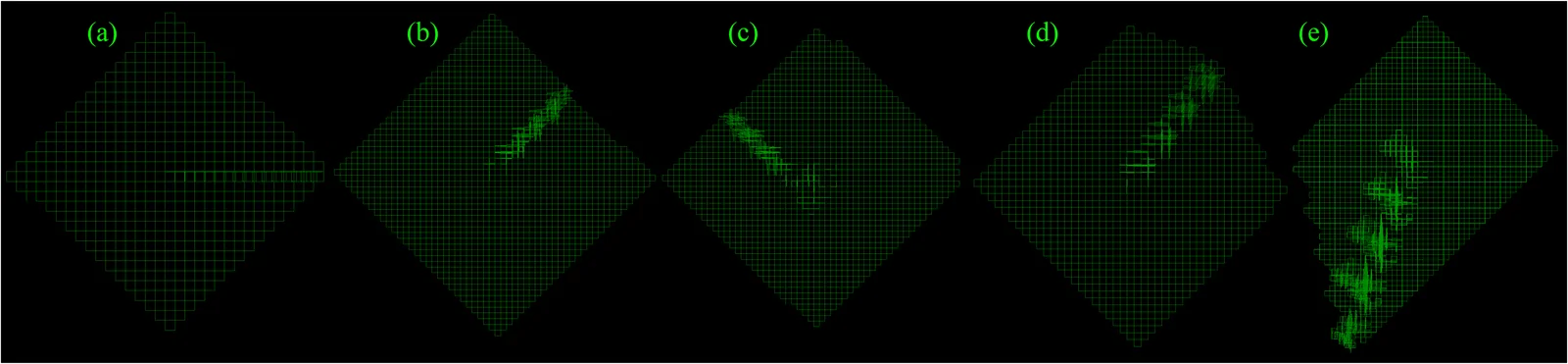
Square building loop worms: (a) 154335, (b) 13543333pop4000, (c) 13434305133pop4000, (d) 1345331145pop3000, (e) 14533311154pop6000 (similar to 13453311154).

**Fig 5:**
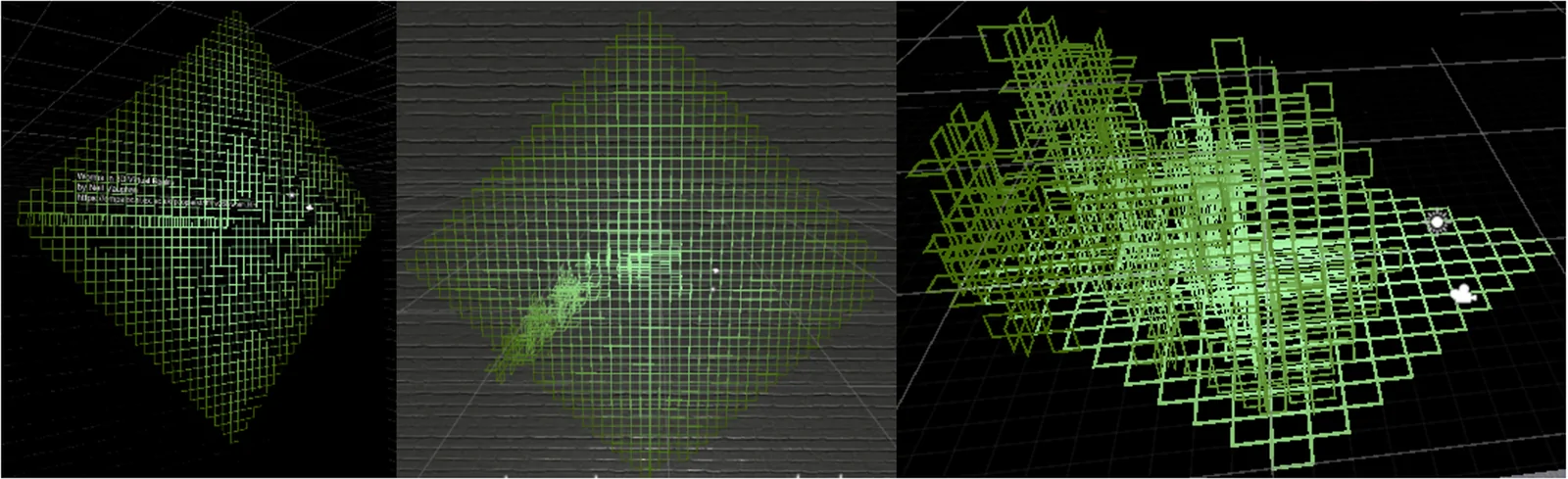
Square builder worm 134534 (and mirror images 314514 and 154335) with 6 rules. The same worm is rendered in C code in Fig 4a. (b) Worm 13434305133 produces a square with a 3D structure. The same worm is rendered in C code in Fig 4c. (c) Worm 13453315451 (and mirror image 45135543134) terminates T at population 5502.

A few looping worm paths grow over time in a two directional infinite square building plane (Fig 4a-e and Fig 5a-b). These are a rarer type of repetitive infinite loop, consisting of perhaps less than 1 The square building worms are mixed with some 3D structure or truss which grows alongside the plane (Fig 4a-e and Fig 5a-b). Viewing the cross section of the 3D structures suggest that these are likely to be infinite shape building loops. Worm 134534 (Fig 4a and Fig 5a), and its mirror images 314514 and 154335 with 6 rules in category L are an example of a square loop which has a horizontal 3D truss that measures one cell in cross section. Worm 13434305133 (Fig 4c and Fig 5b) and it’s mirror images 14343401533 and 41515104355 also grow a square plane with a thicker diagonal truss. A similar thick diagonal truss is present in other 3D square builder worms (Fig 4b, d and e).

There are several false square building worms, which appear to begin building squares, but then change after a few hundred timesteps into a 3D structure which either terminates in category T or enters a one directional loop in category L. An example is worm 13453315451 (and mirror image 45135543134) (Fig 5c which terminates in category T at population 5502 and 11 rules. This initially appears to exhibit a bimodal behaviour because the path resembles a square plane, but it also jumps aperiodically out of the plane where it builds a 3D structure, before then returning to the plane and adding more layers onto the outside edges of the plane.

It can be more difficult to detect square loops algorithmically due to aperiodic changes in direction at the corners. Due to this, some square worms occasionally do not quite meet requirements of the loop detection algorithm. No repetitive looping worms have been identified that grow in all 3 dimensions, such as a cube builder, although there are some chaotic worms which do expand in all 3 dimensions. By chaotic, we mean moving without any foreseeable pattern and no repetition, such that the outcome cannot be known without completing the simulation.

### Loops of record length

Repetitive loops exist in worms in category L. Sometimes the loops are very small or very large. From a recursive search of all worms to a million population, several substantial loops were found (Table 1). The longest loop currently found is worm 14305144543 with a loop of size 296639 (Table 1). Most of the longest loops were identified by automatic detection algorithm, which works by searching for repetitions of any length within the order of rule activations.

**Table 1:** Some of the longest looping patterns in Category L worms.

Worm Code	Category	Loop Size
14305144543	L	296639
13434455503 (Fig 6a)	L	38537
14305540150 (Fig 6b)	L	15716
14305535054	L	14710
14535344113 (Fig 6c)	L	14522
14305535051 (Fig 6d)	L	10995
13430411115	L	10858
10404344501	L	10361
14545355401	L	10361
10403045141	L	9132
10403041130	L	7836

**Fig 6:**
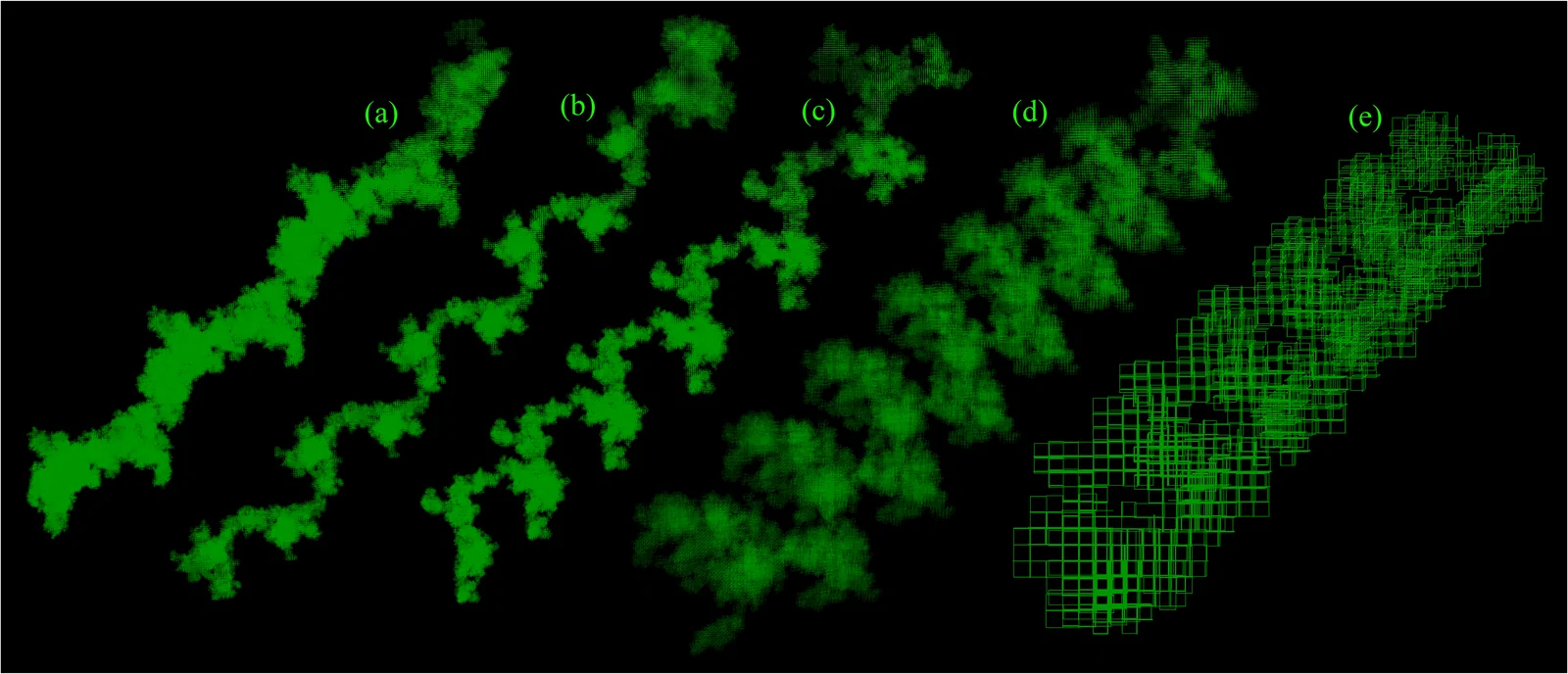
Worms producing long looping paths (a) 13434455503 loop length 38537, (b) 14305540150 loop length 15716, (c) 14535344113 loop length 14522, (d) 14305535051 loop length 10995, (e) 10404311435 with loop length 437.

Longer loops can be detected when worms run to a longer population size. For example, when all worms were run with population limit 5,000, the longest loop was 437 by worm 10404311435 (Fig 6e). The longest loop found with population limit 50,000 run is length 7,602 by worm 14305310141. The longest loop found with population limit 500,000 run is length 38,537 by worm 13434455503 (Fig 6a). The longest loop found with population limit 5,000,000 run is length 296,639 by worm 14305144543. For a loop to be detected by the loop detection algorithm, loops must repeat at least 4 times, so the maximum detectable loop length is limited to a quarter of population limit. A looping section must consist of at least 3 rules. These parameters are sensitive and affect which worms are detected as loops. If the same worms are run again with a higher population limit, some worms classified as R may be re-classified as L with longer loop lengths. Two loops with the same length 10361 are shown in Table 1, these are suspected to be almost mirror image or symmetrical versions of each other and the images of those two worms at length 245,000 appear to confirm this. It appears in Table 1 that 4 of the longest 6 loops found all start with the same set of 5 rules: 14305. A possibility remains that some of the worms currently classified as R may in fact exhibit loops that are so long that it becomes increasingly difficult to discriminate between categories R and L. Recently using high performance computing (HPC), it was identified that several category L worms have loops which start very late, including:

- 13430555141 – Loop of length 2290 detected. Loop starts at population 857272.
- 14305144543 – Loop of length 296639 detected. Loop starts at population 470109 (This is the longest loop detected, a repetition of over a quarter of a million steps).
- 14305135451 – Loop of length 2043 detected. Loop starts at population 332861.
- 13430555411 – Loop of length 16868 detected. Loop starts at population 241264.

### Random rule generation

A run of 15557 random worms was completed with population limit 2100. This is where the rules are selected randomly at runtime from the available paths. The number of duplicates is high when randomly selecting rules, because the same worm is often run many times by chance. In a run of 15557 random worms, 13577 were duplicates, many of the duplicates were short 4 rule worms. This left 1979 (12.7%) unique worms. The minimum number of rules was 4, maximum 11 and average was 5.9. The categories of the worms were, T = 74%, L = 17%, R = 9%. After removing duplicate worms, T = 41%, L = 12%, R = 47%.

### Recursive search results

A recursive search was completed including all worms to a population limit of 12 million. This found 3239 unique worms in categories T, L and R, of which the number of rules in a worm ranges from minimum of 4 to maximum of 11. The mean number of rules in a worm is 10.7, showing that most worms have 11 rules. Of all worms, 88% have 11 rules, 8% have 10 rules and only 4% of worms have between 4–9 rules. The smallest population size of a category T worm is 14, which occurs in only two worms: 1043 and 1430. Currently, the largest observed population size of a worm that terminated category T is population 8,495,866 in worm 13430410141. There are 15 worms remaining unknown after running to 12M and some of these are expected to terminate at even larger population sizes.

### Mirror images

A recursive search of all worms starting with 0, 1, 3, 4 and 5 was completed. Only one worm starts with 0 and it is a category L loop. Worms starting with either 1, 3, 4 or 5 have exactly 9802 worms each, because starting with a 1, 3, 4 or 5 are all exact duplicate mirror images of each other. Therefore all worms starting 3, 4 or 5 can be excluded as they are not unique worms. They all start with 90 degree angles – the only difference is the order that the children worms are run is different in a recursive search, because the rotation of worm is different, so subsequent numbers are ordered differently. This does not affect which worms are run overall, as all paths are checked eventually. Mirror images also occur at deeper levels, such that half of all worms starting 1 are mirror images of other worms starting with 1. For example all worms starting 14 are mirror images of worms starting 15. Within the 9802 worms starting with 1, there are 2776 worms starting with 14, which are mirror images of worms starting 15. There are 663 worms starting 104 which are mirror images of worms starting 105. There are 1459 worms starting 134 which are mirror image of worms starting 135. Due to this, 4899 mirror images are identified (50%) from the 9802, this leaves 4903 potentially unique worms. Other currently undetected mirror image worms are probably present, for example the two worms: 10403045500 and 14054345500, both have exactly the same population size of 1,019,152, and both end with the same 5 rules out of their 11 rules. Another example is three worms all detected as loops with the same loop length of 7836. To quantify the possible extent of duplicates within the 3239 unique worms, the number of unique population sizes within worms terminating T was counted as 1194 out of 1896. The number of unique loop lengths was counted as 157 out of 1348. This proves that the total number of unique worms must be at least 1351 in categories T and L, plus 15 worms in category R, but the total is almost certainly substantially more than this since loops of similar length are not necessarily due to identical or symmetrical worm paths. This is demonstrated by the five worms in Fig 2h-l, of which all 5 worms have the same loop length of 17, but all produce different worm path shapes.

### Proportion of worms in each category

Recursive runs were completed of all worms to various population limits. The worms have been classed according to their outcome. Mirror image and reflection worms were removed. Category N worms are not included, because they are incomplete worms and all their children are already included separately and no category N worms with 11 rules have been found yet. The proportion of worms in each category is summarised in Table 2. As the population limit increases, the number of category R worms decreases. When population limit increases from 5,000–50,000, as additional 4 unique worms were found. This is because some of those worms were previously in category R at 5,000 but at higher population, those worms carried on running and became category N, so then their children worms were also run which were not run in the 5,000 version. Worms usually need a high number of rules to remain in category R. Of the 262 unknown category R worms in the 50,000 population limit, the minimum number of rules is 10, but only 5 worms have 10 rules, and all 257 others (98%) have 11 rules. Likewise with 500,000 population limit, out of the 72 category R worms, only one worm has 10 rules, all 71 other worms have 11 rules. Some worms start by moving un-repetitively but then become loops later on. Of the 1237 category L worms at 50,000 population limit, 110 are detected as L quite late at 45,000 population. Also some worms such as shape builders are harder to detect as loops until they become larger, due to increased complexity in the structure of their repetitive patterns.

**Table 2:** Proportion of worms in each category.

Population limit	Unique Worms	T	L	R
5,000	3234	1418	868	948
50,000	3238	1739	1237	262
500,000	3239	1830	1337	72
3,000,000	3239	1866	1342	31
12,000,000	3239	1876	1348	15
53,500,000	3239	1882	1352	5

### Rule activation counts

To identify which rules are activated most often, the number of times that each of the 32 possible binary rules were activated was counted during a recursive run of all 9822 worms, starting with worm 1, up to 500,000 population limit, including mirror worms within children of worm 1. Of the 2^5^ = 32 possible binary rules, 21 are activated but 11 rules are never activated in any worms starting with 1. Of the 11 unused rules, 7 can be explained. The rule 111111 has no paths so it’s isn’t possible as this will automatically terminate. A further two rules: 011100 and 001011 can never be activated as they require there to be a pre-existing straight path of length >1 which cannot exist except in worm 0. There are 5 rules which have 4 paths, but only one of those is used in worms starting with 1: 011000 because that always occurs as the second rule straight after rule 1, and a worm can only contain one rule with 4 paths, because rules with even number of paths only occur when passing the origin. Three of the other 4 path rules would occur only in worms starting 3–5 respectively. The 4 path rule 101000 is clearly impossible because a worm cannot arrive back at the origin from the opposite of starting direction without other paths having being used. It remains unknown why the other four rules have not yet been seen to occur: 001111, 011101, 011110 and 101011. These all have 2 paths available and are all mirror images of each other, although rules 111100 and 011011 are also their mirror image and they do occur. Perhaps these four rules would be used in mirror image worms starting with 3–5, but this hasn’t been confirmed yet. Perhaps this is partially because overall, rules with 2 paths occur much fewer times than others: 101101 triggered 340 times, compared to 001000 triggered 88,048,599 times. Rules 011001 and 011010 both activated exactly 14,923,776 times, probably due to mirror image worms within children of worm 1.

### Worms terminating at a high population size

**Fig 7:**
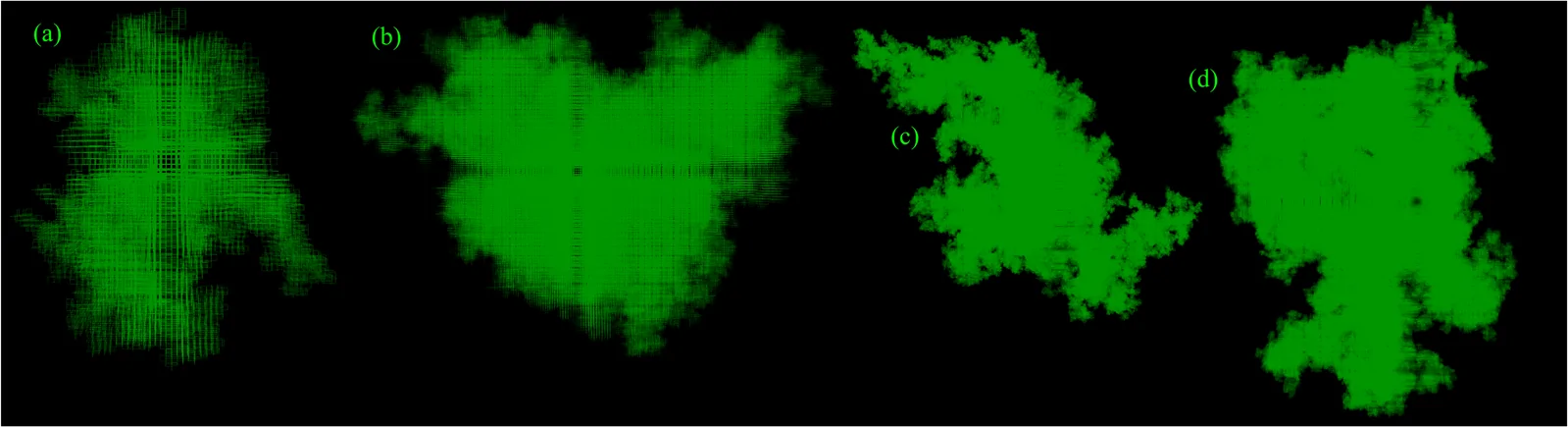
High population size category T terminating worms: (a) The worm 10403030113 terminated in category T with population 99,502 and 11 rules. (b) Worm 15433350401 (and mirror image 14533340501) terminates T at 2,511,942. (c) Worm 10403041453. Termination code T. Rules 11. Population 7,304,810. (d) Worm 13430410141 terminates T at 8,495,866 population.

The largest worm to terminate in category T under 100,000 population is 10403030113 with population 99,502 (Fig 7a). Overall, 18 worms terminate category T between 500,000 and 1 million. A further 28 worms terminate category T between 1 million and 10 million. Some examples are the worms 14533340501 and it’s mirror image 15433350401 (Fig 7b) terminate T at 2,511,942. Another example is 10403041453 which terminated with population 7,304,810 (Fig 7c). Worm 13430410141 terminated at population size 8,495,866 (Fig 7d).

### Worms with unknown outcome beyond 12 million population

**Fig 8:**
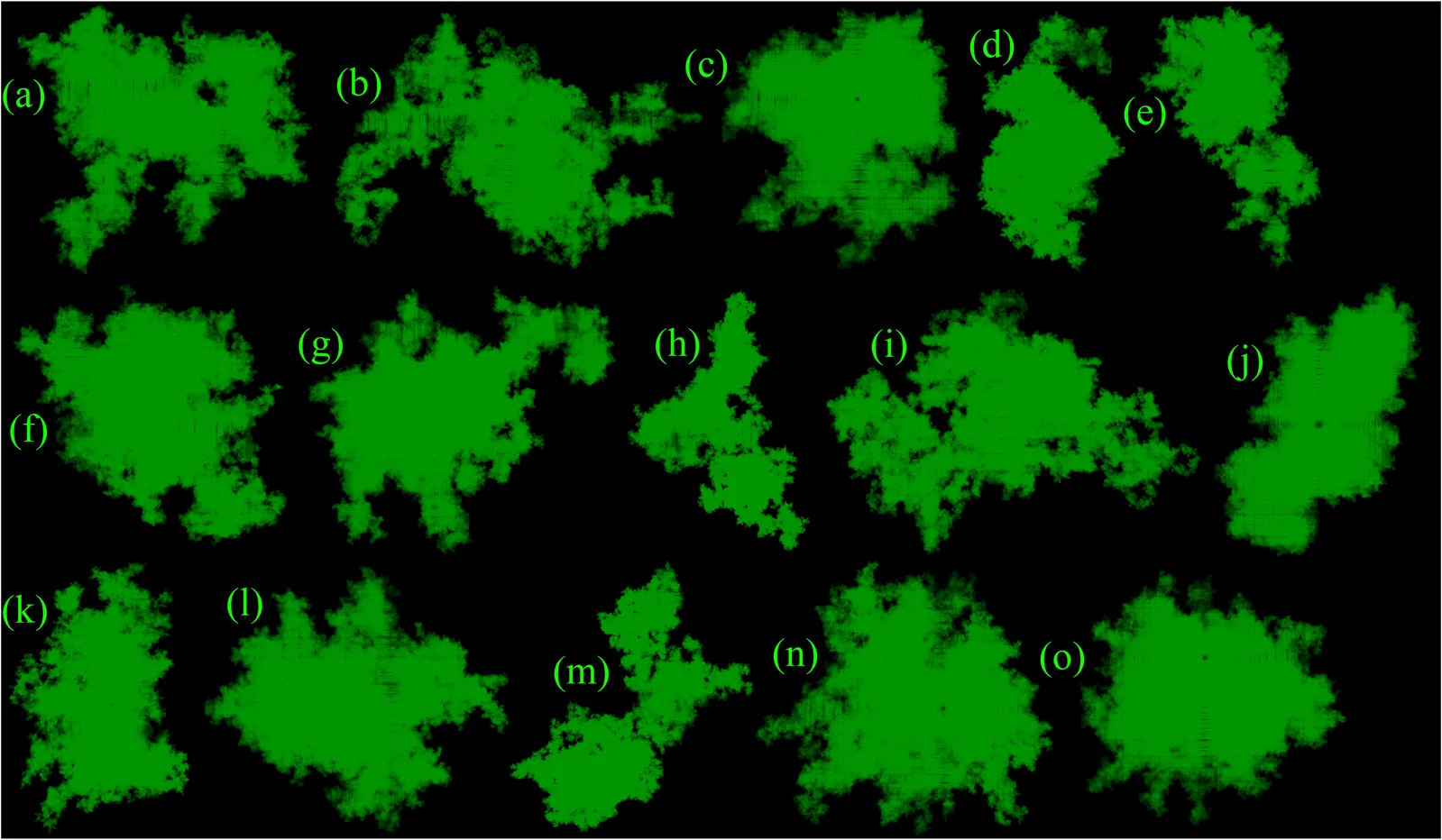
The only 15 worms that continue running chaotically in category R beyond 12 million population. This image was recorded at 5 million population. The worm codes are in the order: (a) 10403030151, (b) 10403031514, (c) 10403041415, (d) 10403045304, (e) 13430511544, (f) 13430514101, (g) 13430551315, (h) 14054330151, (i) 14054331514, (j) 14054345304, (k) 1430511544, (l) 14305130053, (m) 14305141553, (n) 14305535431, (o) 14305540401.

**Fig 9:**
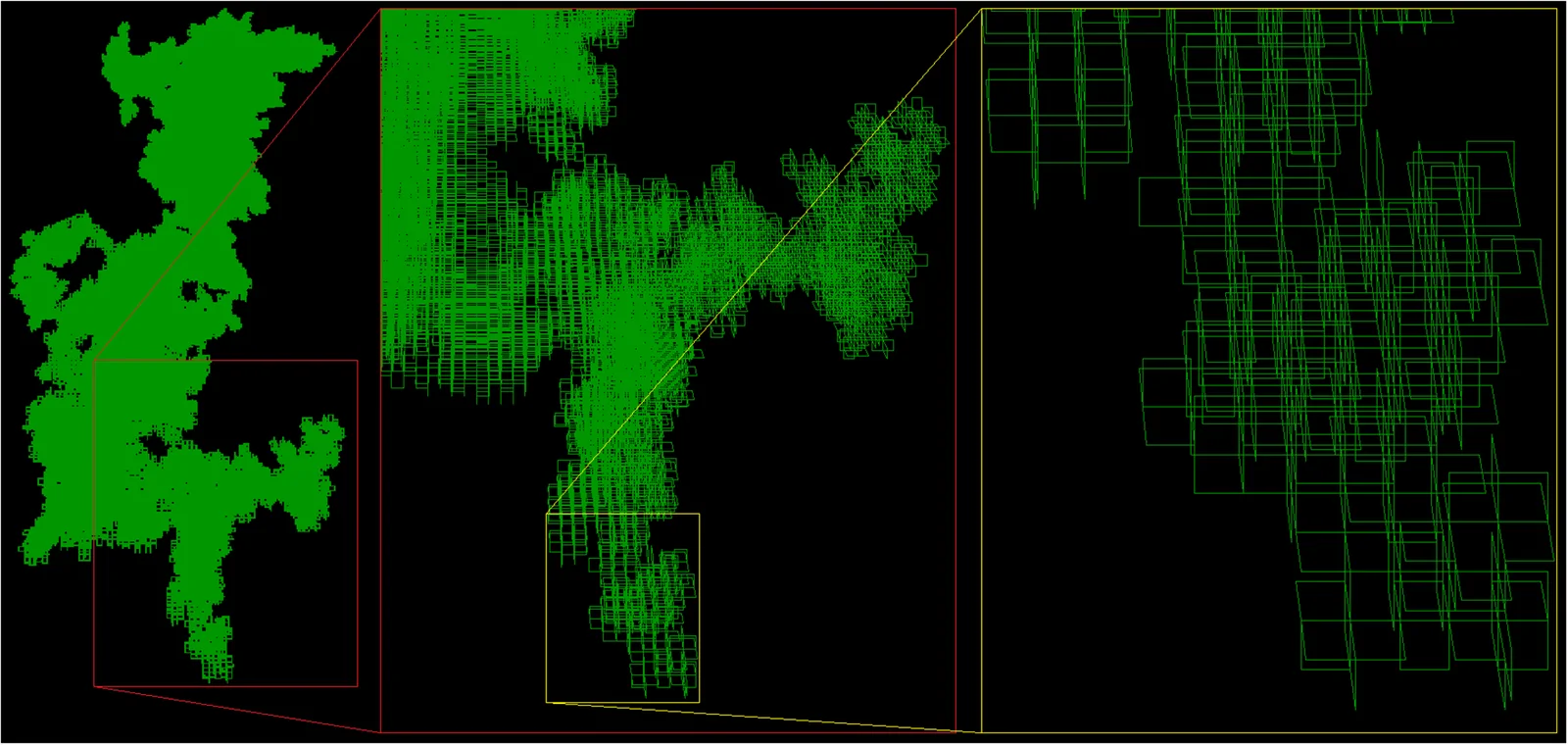
Different levels of zoom demonstrating the emerging complexity. Worm 10403030151 which eventually terminated in category T on high performance computer with population 66,348,894. This worm has a ‘twin’ worm 14054330151 (Fig 8h) which also terminates T with exactly the same population size.

There are only 15 worms which continue running in category R beyond 12 million population (Fig 8). These fifteen worms are shown in Fig 8 at a population of 5 million. Five of these fifteen worms begin with the same set of five rules: 14305, and these five rules also appeared to produce several of the longest loops found (Table 1). The complexity within one chaotic worm is shown in Fig 9 with additional zoom. Recently, using high performance computing (HPC), these 15 worms were run to larger populations and 10 of these 15 worms are now solved, which leaves only 5 worms with currently unknown outcome: 13430551315 continues in category R beyond 86.67M, 14305141553 continues in category R beyond 69.33M, 13430514101 continues in category R beyond 68.53M, 14054345304 continues in category R beyond 54.90M and 10403045304 continues in category R beyond 53.50M population. These final 5 worms still have unknown outcome and continue in category R. Worm 14305540401 terminated T with population 61,119,698, and worm 14305130053 terminated T with population 23,470,588.

## Discussion

This research makes several novel contributions. (a) A new 3D grid is proposed for modelling worm paths. (b) Analysis is presented from modelling all 3239 unique worms that emerge from this new grid. (c) The first modelling of 3D worm paths is included with immersive virtual reality (VR) to adequately visualise higher dimensionality. (d) The Virtual Reality (VR) Google cardboard Android APK file has been developed and made available which runs on most android smartphones, enabling other researchers to experiment with and visualize these 3D worm paths. (e) A JavaScript version of 3D worms has been developed, enabling researchers to view the 3D worm paths in any web browser on any platform from windows, apple or smart devices. (f) A new classification model is proposed for determining the outcome of worm paths into categories N, I, T, L and R. (g) Various techniques for automatic detection of looping worms was developed and described. (h) A recursive algorithm was proposed for recursively searching through all child worms in a tree structure and automatically logging all results. (h) Some major branches of worms were identified which are mirror images and reflections of other worms. (i) Images of interesting worms are presented including complex loops (Fig 6) and fifteen worms that continue running beyond 12 million population, five of which still have unknown outcome (Fig 8).

## Conclusion

**Fig 10:**
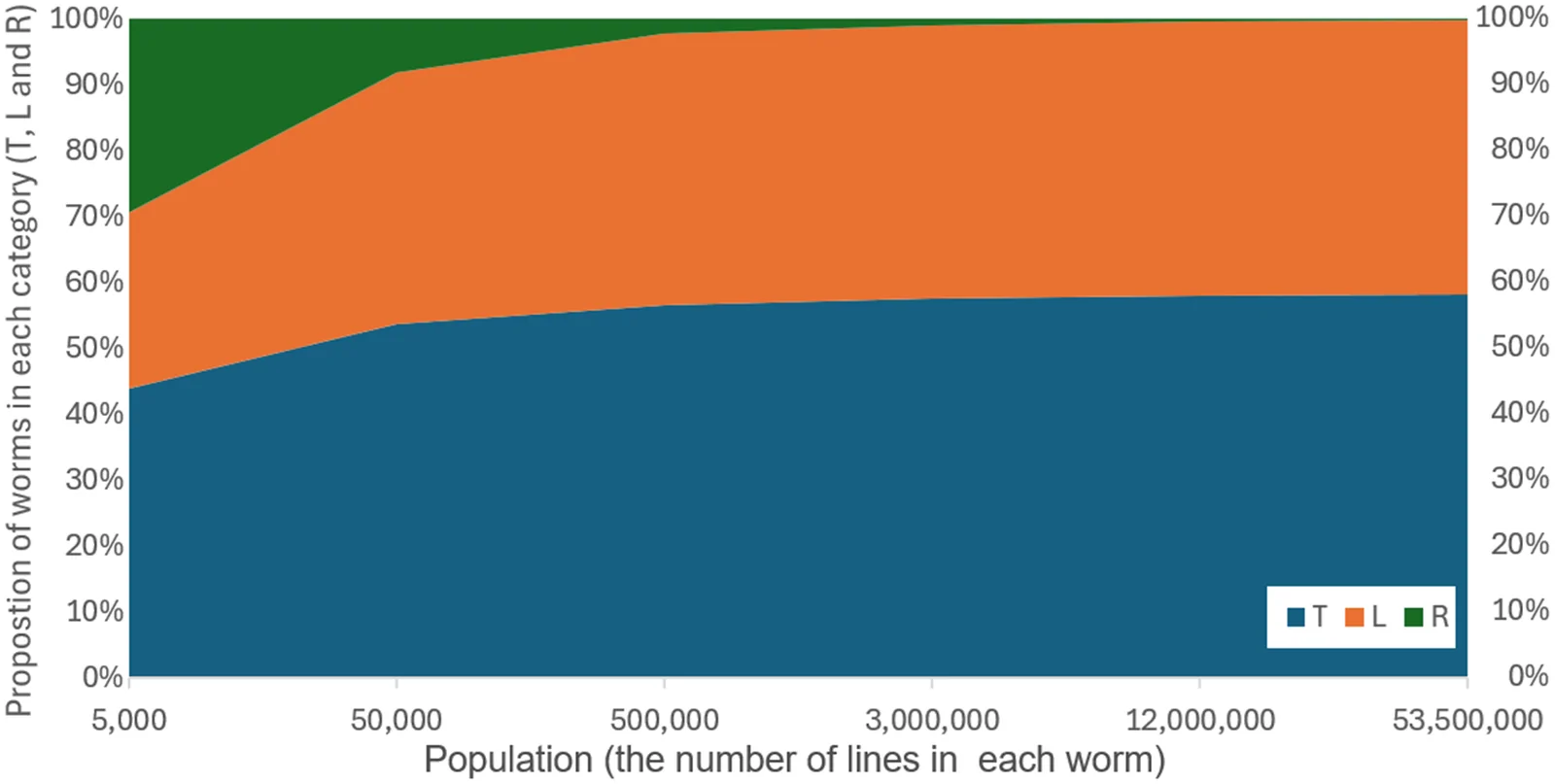
Stacked area chart. This shows that when worms are run for a longer time (to a larger population, meaning a larger number of lines per worm), the proportion of worms in each category changes, and finally, 58% of all worms end as category T (terminated), 42% of worms end as category L (looping repetition) and only about 0.1% of worms (5 individual worms) remain in category R (still running with unknown outcome) beyond 53.5 million which was as far as the current hardware could explore. It is conjectured that no worms on any grid continue to run in category R infinitely.

The proposal of this new 3D grid for modelling worms, demonstrates that the initial method of modelling worms on a triangular hexagonal grid is not the only approach. There are many other grid types that haven’t been investigated yet, perhaps an infinite number of grids. For example other grids include a 3D orthogonal cube grid allowing diagonals, which would have 14 paths per step. An extreme example would be a 2D grid with 360 paths per step, with 1 degree between each path. An orthogonal 10 dimensional grid would have 20 paths per step. The greater the number of paths per step, the greater the number of possible worms in general, and the longer the worms can stay running in category R. Findings from worms on one grid type appear to remain true in other types of grid. One fact which would appear to always remain true, regardless of grid type, is that as population increases, the number of worms remaining running in category R decreases, as shown in Fig 10. It appears that on any grid style including n-dimensionality, no worms seem to continue running chaotically forever in category R, because they tend to 0 as population increases, as worms become category L loops or terminate T at the origin, future work may search for a mathematical proof of this, as counterexamples cannot be shown in finite time. This research has presented images to highlight several of the most interesting worms on this 3D grid, including (1) the final five worms with unknown outcome that continue running in category R beyond 53.5 million timesteps, (2) worms that appear to switch back and forth bimodally between a 2D plane and a 3D structure.

## Supporting information

S1 File Javascript Code 3D implementation. (HTM)
