# Supplementary material for: Mathematical model for worm path simulation in 3D
Source: PLoS One. 2026 Sep 11;21(9):e0358065. doi: 10.1371/journal.pone.0358065 (PMC13568494; doi:10.1371/journal.pone.0358065)
Supplement: Supplementary file 1 [file pone.0358065.s001.htm]

Worm Simulator Neil Vaughan


# Worm Simulator in 3D

Press g to generate a random worm.  
Press r or F5 to reset all to blank.  
Type rule numbers from 0-5 to program a new worm. For example worm code: 1034 (population 22).  
Worm code:  


  
© Neil Vaughan 2021
