# Supplementary material for: Mathematical model for worm path simulation in 3D
Source: PLoS One. 2026 Sep 11;21(9):e0358065. doi: 10.1371/journal.pone.0358065 (PMC13568494; doi:10.1371/journal.pone.0358065)
Supplement: Supplementary file 2 [file pone.0358065.s002.docx]

The research presented here looks at possible 3D worm paths on an orthogonal grid. Previous work has focused exclusively on 2D grids with various tilings. Such a grid better reflects the spatial complexity of the space that worms occupy and is thus of importance for understanding their movement. The author explores how a deterministic, rule-based algorithm with <= 32 parameters controls the worm movements. Worms are classified as Terminated, Looping, or Running depending on the outcome of the simulation. The authors present interesting worms and set the stage for future work to explore the types of worms in this model. Additionally, virtual reality and 3D visualization is created to allow for immersive exploration of the paths. The JavaScript is helpful for understanding some of the algorithm.

The paper lacks technical descriptions of many aspects, and those that are provided do not offer sufficient clarity. Examples include “chaotically” and “population”. After reviewing, I still do not understand what a “rule” is. It would seem to me that for the worm that immediately completes a square to return to the origin, that when it returns to the origin, it must select a new rule that continues it on its path since 4 such paths are available at that time. That is how I read “The worm continues in this way and if a situation is encountered where no paths are available, the worm terminates.”

Another key limitation of the presented work is lack of figures that summarize the analyses that are suggested in the text. At minimum, summaries of how many worms end up in each category should be shown. Given how central the idea of “population” and/or “time” is, showing how this evolves over time would be useful. Finally, loop length.

Overall, this paper has much to improve upon to make it a stronger contribution to the field and to inform readers.

1. The exposition of various aspects of the model and its analysis are provided in a convoluted ordering, making it difficult to comprehend for a reader new to the field.
2. The worm codes are not explained. Is there a special meaning to “14305144543” for example that can be parsed by readers? If not, summaries across the various grouping of worms would provide more informative visualizations. For example, rather than Table 1, a histogram of Loop Size over all category L worms would be more informative.
3. This also raises another concern: all the figures are various renderings of the paths that worms take. There are no variations that provide a greater context for these results as the author has done in previous publications.
4. “whenever the same situation is encountered.” I have an intuition about what this means for the worm’s rules, but it would help to state this more explicitly. I interpret it to mean “whenever the same set of available paths is encountered.”
5. It might be beyond the author’s control as the field may have already set this, but the 0-5 “paths” and the numbered edges of the worm’s overall path feels semantically overloaded.
6. The concept of “population” is not defined. For someone outside this field, this is an unintuitive use of this word.
7. Is there a technical definition for “chaos” in this field? Providing one here would help readers understand the precise point the author is making.
8. The language of the abstract and conclusion is too informal for a research paper. To give specifics: 1) Use of contractions. 2) Describing worms as “interesting” rather than a more technical term such as “unique” or “outlier”.
9. I believe a 3D orthogonal grid with diagonals allowed will either provide 26 or—if <1,1,1>-type diagonals are excluded—18 paths are allowed. That’s what my Rubik’s cube says.
