# Supplementary material for: Mathematical model for worm path simulation in 3D
Source: PLoS One. 2026 Sep 11;21(9):e0358065. doi: 10.1371/journal.pone.0358065 (PMC13568494; doi:10.1371/journal.pone.0358065)
Supplement: Supplementary file 3 [file pone.0358065.s003.docx]

***WormsReview.doc***

The research presented here looks at possible 3D worm paths on an orthogonal grid. Previous work has focused exclusively on 2D grids with various tilings. Such a grid better reflects the spatial complexity of the space that worms occupy and is thus of importance for understanding their movement. The author explores how a deterministic, rule-based algorithm with <= 32 parameters controls the worm movements. Worms are classified as Terminated, Looping, or Running depending on the outcome of the simulation. The authors present interesting worms and set the stage for future work to explore the types of worms in this model. Additionally, virtual reality and 3D visualization is created to allow for immersive exploration of the paths. The JavaScript is helpful for understanding some of the algorithm.

Thank you for this summary description

The paper lacks technical descriptions of many aspects, and those that are provided do not offer sufficient clarity. Examples include “chaotically” and “population”.

I have now added definitions of both chaotically and population in multiple places in the manuscript.

After reviewing, I still do not understand what a “rule” is. It would seem to me that for the worm that immediately completes a square to return to the origin, that when it returns to the origin, it must select a new rule that continues it on its path since 4 such paths are available at that time. That is how I read “The worm continues in this way and if a situation is encountered where no paths are available, the worm terminates.”

Yes this is correct. An example of a complete rule is something like : 10403030151. Each number represents which path (from 0 to 5) should be chosen at each decision point. The first path would be path 1, then when another choice is needed, path 0 would be chosen as that is the next number. I have now modified the text to clarify this.

Another key limitation of the presented work is lack of figures that summarize the analyses that are suggested in the text. At minimum, summaries of how many worms end up in each category should be shown. Given how central the idea of “population” and/or “time” is, showing how this evolves over time would be useful. Finally, loop length.

I agree this would help to visualise and clarify. Added to manuscript: “Fig. 10. Stacked area chart showing that when worms are run for a longer time (to a larger population with a larger number of lines per worm), the proportion of worms in each category changes, and finally, 58% of all worms end as category T (terminated), 42% of worms end as category L (looping repetition) and only about 0.1% of worms (5 individual worms) remain in category R (still running with unknown outcome) beyond 53.5 million which was as far as the current hardware could explore.”

Overall, this paper has much to improve upon to make it a stronger contribution to the field and to inform readers.

1. The exposition of various aspects of the model and its analysis are provided in a convoluted ordering, making it difficult to comprehend for a reader new to the field.
2. The worm codes are not explained. Is there a special meaning to “14305144543” for example that can be parsed by readers? If not, summaries across the various grouping of worms would provide more informative visualizations. For example, rather than Table 1, a histogram of Loop Size over all category L worms would be more informative.

Yes the numbers such as 10403030151 are examples of rules which produces a specific worm path. Each number represents which path (from 0 to 5) should be chosen at each decision point. The first path would be path 1, then when another choice is needed, path 0 would be chosen as that is the next number. Recording these makes the worms reproducible and enables counting the unique worms.

3. This also raises another concern: all the figures are various renderings of the paths that worms take. There are no variations that provide a greater context for these results as the author has done in previous publications.
4. “whenever the same situation is encountered.” I have an intuition about what this means for the worm’s rules, but it would help to state this more explicitly. I interpret it to mean “whenever the same set of available paths is encountered.”

I agree, manuscript updated to replace ‘situation’ with ‘set of available paths.’

5. It might be beyond the author’s control as the field may have already set this, but the 0-5 “paths” and the numbered edges of the worm’s overall path feels semantically overloaded.
6. The concept of “population” is not defined. For someone outside this field, this is an unintuitive use of this word.

Added to section 1.1: “Population means the number of lines in the worm.”

7. Is there a technical definition for “chaos” in this field? Providing one here would help readers understand the precise point the author is making.

Added a definition of ‘chaotic’: “By chaotic, we mean moving without any foreseeable pattern and no repetition, such that the outcome cannot be known without completing the simulation.”

8. The language of the abstract and conclusion is too informal for a research paper. To give specifics: 1) Use of contractions. 2) Describing worms as “interesting” rather than a more technical term such as “unique” or “outlier”.

Removed the contractions including replaced “haven’t” with “have not.” Replaced ‘interesting’ with ‘unique outlier.’

9. I believe a 3D orthogonal grid with diagonals allowed will either provide 26 or—if <1,1,1>-type diagonals are excluded—18 paths are allowed. That’s what my Rubik’s cube says.

This may be correct and interesting for future research including diagonals on a cube. That number 26 would also need to be reduced since retracing the path is not allowed, so it may have a maximum of 25 available path choices. Currently in this 3D cube research, diagonals were not allowed. We have previously explored diagonals on a 2D grid, and would be interesting to look into that in 3D. Even with 6 or 8 paths the number of possible worms is huge, so with 26 paths, it would only be possible to explore a microscopic proportion of the astronomical number of potential worms.

3. Thank you for stating the following financial disclosure:
This research was funded by the Science and Technology Program of Guangxi, China (No. 2023AB08158)

This is not correct. In the manuscript, the acknowledgement states this: “The research was supported by the Royal Academy of Engineering (RAEng) through a research fellowship awarded to N. V.”

Please state what role the funders took in the study. If the funders had no role, please state: "The funders had no role in study design, data collection and analysis, decision to publish, or preparation of the manuscript."
If this statement is not correct you must amend it as needed.
Please include this amended Role of Funder statement in your cover letter; we will change the online submission form on your behalf.

I have added this into the manuscript under acknowledgements: “The funders had no role in study design, data collection and analysis, decision to publish, or preparation of the manuscript.”

4. We note that Figure(s) 1, 2, 3, 4, 7 in your submission contain copyrighted images. All PLOS content is published under the Creative Commons Attribution License (CC BY 4.0).

This does not apply – All figures in this manuscript are original and unpublished, custom-made for this publication only.

5. Please note that PLOS One has specific guidelines on code sharing for submissions in which author-generated code underpins the findings in the manuscript. In these cases, we expect all author-generated code to be made available without restrictions upon publication of the work. Please review our guidelines at <https://journals.plos.org/plosone/s/materials-and-software-sharing#loc-sharing-code> and ensure that your code is shared in a way that follows best practice and facilitates reproducibility and reuse.

As requested, the author has now shared all the code in public domain accessible through reference [12], including .APK android file, JavaScript, and C code, all unpublished and custom made for this publication only.

6. Please provide a complete Data Availability Statement in the submission form, ensuring you include all necessary access information or a reason for why you are unable to make your data freely accessible. If your research concerns only data provided within your submission, please write "All data are in the manuscript and/or supporting information files" as your Data Availability Statement.

The author has shared all the code in public domain accessible through reference [12].

7. Thank you for stating the following in the Acknowledgments Section of your manuscript:
This research was supported by the Royal Academy of Engineering (RAEng) through a research fellowship awarded to N. V.

We note that you have provided funding information that is not currently declared in your Funding Statement. However, funding information should not appear in the Acknowledgments section or other areas of your manuscript. We will only publish funding information present in the Funding Statement section of the online submission form.
Please remove any funding-related text from the manuscript and let us know how you would like to update your Funding Statement. Currently, your Funding Statement reads as follows:
Royal Academy of Engineering (RAEng) Research Fellowship to NV

Please include your amended statements within your cover letter; we will change the online submission form on your behalf.

Please include this funding acknowledgement:

“This research was supported by the Royal Academy of Engineering (RAEng) through a research fellowship awarded to N. V. The funders had no role in study design, data collection and analysis, decision to publish, or preparation of the manuscript.”

8. [Please update your submission to use the PLOS LaTeX template. The template and more information on our requirements for LaTeX submissions can be found at http://journals.plos.org/plosone/s/latex.](https://eur03.safelinks.protection.outlook.com/?url=http%3A%2F%2Ftrack.editorialmanager.com%2FCL0%2Fhttp%3A%252F%252Fjournals.plos.org%252Fplosone%252Fs%252Flatex.%2F1%2F010f019e393e1292-c6933d54-c4b7-481e-9a1e-0e0593369587-000000%2FRPbUtMhFefRxz2qW8ksBU2-Yx9B92RcpGyEs_rY53Qg%3D258&data=05%7C02%7Cn.vaughan%40exeter.ac.uk%7C95b650e11d0e4eefadde08deb4920147%7C912a5d77fb984eeeaf321334d8f04a53%7C0%7C0%7C639146736660505318%7CUnknown%7CTWFpbGZsb3d8eyJFbXB0eU1hcGkiOnRydWUsIlYiOiIwLjAuMDAwMCIsIlAiOiJXaW4zMiIsIkFOIjoiTWFpbCIsIldUIjoyfQ%3D%3D%7C0%7C%7C%7C&sdata=sC35Nr6yan45%2F0gP6mDx%2BBCj399YEseyUwRVY7hsGiE%3D&reserved=0)

9. We note that Figure(s) 1-9 in your submission contain copyrighted images. All PLOS content is published under the Creative Commons Attribution License (CC BY 4.0), which means that the manuscript, images, and Supporting Information files will be freely available online, and any third party is permitted to access, download, copy, distribute, and use these materials in any way, even commercially, with proper attribution. For more information, see our copyright guidelines: <http://journals.plos.org/plosone/s/licenses-and-copyright>.

N/A all figures are original and unpublished.

10. [Please include captions for your Supporting Information files at the end of your manuscript, and update any in-text citations to match accordingly. Please see our Supporting Information guidelines for more information: http://journals.plos.org/plosone/s/supporting-information.](https://eur03.safelinks.protection.outlook.com/?url=http%3A%2F%2Ftrack.editorialmanager.com%2FCL0%2Fhttp%3A%252F%252Fjournals.plos.org%252Fplosone%252Fs%252Fsupporting-information.%2F1%2F010f019e393e1292-c6933d54-c4b7-481e-9a1e-0e0593369587-000000%2FEZfVwhLFlAuYQKo1XmlVJUJMbo03bYUVUeNhyxiHHfs%3D258&data=05%7C02%7Cn.vaughan%40exeter.ac.uk%7C95b650e11d0e4eefadde08deb4920147%7C912a5d77fb984eeeaf321334d8f04a53%7C0%7C0%7C639146736660518465%7CUnknown%7CTWFpbGZsb3d8eyJFbXB0eU1hcGkiOnRydWUsIlYiOiIwLjAuMDAwMCIsIlAiOiJXaW4zMiIsIkFOIjoiTWFpbCIsIldUIjoyfQ%3D%3D%7C0%7C%7C%7C&sdata=JJa8ryIM05XXxWx%2BWmyzGq6DFI9NpGQHhoZdqxdCrSI%3D&reserved=0)

11. If the reviewer comments include a recommendation to cite specific previously published works, please review and evaluate these publications to determine whether they are relevant and should be cited. There is no requirement to cite these works unless the editor has indicated otherwise. 

5. Review Comments to the Author

Reviewer #1: Re #3: The author did not provide all code used in this work. Furthermore, the code is not publicly available, though I do not consider that an issue at the review stage.

As requested, the author has now shared all the code in public domain accessible through reference [12], including .APK android file, javascript, and C code, all unpublished and custom made for this publication only.

Please see my attached comments for further info regarding the Major Revision decision.

Reviewer #2: This paper extends the classical Paterson worm model from 2D grids to a 3D cubic orthogonal lattice. Through exhaustive recursive simulation, the author reports 3239 unique worms and classifies their outcomes into terminating, looping, and running categories. However, several concerns limit the strength of the contribution. The reliability of the reported results is not independently verified.

This is an original work, and no other researchers have worked on this topic, so there is no independent verification. However the code is fully shared which fully enables any future researchers to replicate and independently verify or build on this further.

Some theoretical claims, such as the 13-rule upper bound, are not supported by rigorous mathematical proof.

I agree, in response, we have now removed “with a proof” as this is not an official proof.

A description of the proof is given by the author very clearly and precisely and this is strong enough to conclusively prove that 13 is the maximum possible rule upper bound, without any doubt, this is an original new finding (included in manuscript):

It is shown with a proof that no worms in this 3D grid can use more than 13 rules. Worms have 6 binary sensors but path 2 is always unavailable, which leaves 25 possible rule choices. In theory up to 32 binary rules are available, because there are maximum of 32 possible binary rule representations from 00000 to 11111. However, half of these rules have an even number of paths available, so only 2 of these can be used per worm, because an even number of available paths can only occur when passing through the origin, which cannot happen more than twice. Of the remaining 16 rules with an odd number of paths, 5 of those rules have only 1 path which are automatically selected without rule setting. One rule has 5 paths which is the first rule, it is restricted to 1 and it does count as a rule. Additionally, this leaves 10 rules with 3 paths. In addition a worm could use 2 rules with an even number of paths: 4 and 2 which can occur.

This shows that the total maximum number of rules that could be used by one worm is restricted to 13.

The biological motivation and application for extending the model to 3D requires further clarification.

This is certainly a bio-inspired model but sits at the interface of biology and mathematics. Like the original Paterson’s worm in 2D, there are some differences between this abstract mathematical model and the actual biological workings in-vivo real worms. However the main take-aways from proposing and analysing a model such as this, are still profound and meaningful, as stated in the manuscript, that this work clarifies that “Simple rules result in surprisingly complex and intricate movements.” And “Images and interesting observations are presented demonstrating complex 3D patterns that emerge.”

1. The biological motivation for extending the worm foraging problem to 3D should be explained more clearly. It is unclear what real-world biological or physical process corresponds to discrete orthogonal jumps on a 3D cubic lattice. How can a worm move through 3D space without any supporting medium or substrate? Grounding the model in a concrete real-world scenario would strengthen the motivation for this research.

Worms are not expected to “move through 3D space without any supporting medium or substrate.” In real worms, they burrow underground, worms can clearly burrow tunnels horizontally as well as vertically, so in-vivo worms exist in a 3D soil substrate. From this perspective the original 2D Paterson’s worms seems very restrictive as only surface dwelling creatures are restricted to 2D movement, worms which exist within the underground soil are moving in 3D.

2. All results in this paper are based on a single fixed set of hyperparameters. For example: the population limit (53.5M steps), the loop detection thresholds (≥4 repetitions, ≥3 rules per cycle), and the manually identified mirror symmetries. A sensitivity analysis showing how the reported numbers change under different parameter choices would be important for assessing the robustness of the conclusions.

The mentioned population limits were only due to limitations of hardware and time, ultimately it is unknown if these worms are infinite or not, if they are then the end can never be reached. In this research we tried to reach the end but the hardware available only allowed up to 53.5M steps, which is already a massive leap from previous research which approached only 10M or 12M steps.

3. The purpose of the random search experiment at population limit 2,100 is unclear. Can the resulting T/L/R proportions be compared against a recursive search at the same population limit? Such a direct comparison would help clarify what this experiment contributes beyond the recursive results.

The reviewer raises a correct point, that the random search experiment was essentially ‘superseded’ later on by our more extensive recursive search. Therefore, it is true that all worms found in the random search were also run in the full recursive search. Therefore the random search didn’t explore anything that wasn’t already covered. However, since the random search was completed first, the authors mentioned that it was done, and one interesting finding from the random search was that 1979 worms were found, of the 3239 unique worms (about 61% of all worms) were found in the random search.

4. In Section 1.4, the author claims "It is shown with a proof that no worms in this 3D grid can use more than 13 rules." However, the argument that follows is an informal verbal explanation rather than a rigorous mathematical proof. A formal proof structure is needed to support this claim.

I agree, in response, we have now removed “with a proof” as this is not an official proof.

A description of the proof is given by the author very clearly and precisely and this is strong enough to conclusively prove that 13 is the maximum possible rule upper bound, without any doubt, this is an original new finding (included in manuscript):

It is shown with a proof that no worms in this 3D grid can use more than 13 rules. Worms have 6 binary sensors but path 2 is always unavailable, which leaves 25 possible rule choices. In theory up to 32 binary rules are available, because there are maximum of 32 possible binary rule representations from 00000 to 11111. However, half of these rules have an even number of paths available, so only 2 of these can be used per worm, because an even number of available paths can only occur when passing through the origin, which cannot happen more than twice. Of the remaining 16 rules with an odd number of paths, 5 of those rules have only 1 path which are automatically selected without rule setting. One rule has 5 paths which is the first rule, it is restricted to 1 and it does count as a rule. Additionally, this leaves 10 rules with 3 paths. In addition a worm could use 2 rules with an even number of paths: 4 and 2 which can occur.

This shows that the total maximum number of rules that could be used by one worm is restricted to 13.

5. The quality of all figures in the paper is insufficient. The individual worm structures are difficult to distinguish. The figures should be reworked at higher resolution and clarity to convey the intended information.

I agree it would be great to increase to higher resolution. Currently, it was not possible to generate perfect images, due to requiring incredible high detail, such as over 86 million lines in a single worm. To improve these, the images have been now rendered in three separate rendering tools: Unity, Java and C. The best images have been selected giving the highest clarity of detail. Figure 9 also contains two inner zoom panels, to show how the details increase further with unprecedented scale. Figure 8 aims to show the fully zoomed out versions, so the fine details are not visible, but it does demonstrate the outline shape of each pattern nicely.

Reviewer #3: The manuscript presents a computational study on a 3D orthogonal grid, framed as simulations of worm paths. The author extends previous 2D triangular/hexagonal grid models to 3D, enumerating 3239 unique worm configurations and classifying their outcomes.

Thank you for this description

The problem of this work which cannot be easily fixed is its narrow scope. All the experiments performed are held in very particular conditions (e.g., a reader can see a lot of arbitrary numbers as parameter values which are not justified). The author does not place his work into a broader scientific scope and fails to generalize his results.

Abstract, introduction has been updated. Also a Figure 10 has now been added. This will help to place this work into a broader scientific scope and to generalize these novel results. Abstract modified to state: “Simple rules result in surprisingly complex and intricate movements. Images and interesting unique outlier observations are presented demonstrating complex 3D patterns that emerge.”

In fact, the author's work falls into the scientific area of self-avoiding walks (SAW) which is not mentioned anyhow. Based on the fact that the references list is rather short (14 papers), I assume that the author was not thorough enough when analyzing the corresponding works of other researchers.

It was known by the authors that other wider research such as self-avoiding walks (SAW) are existing. However, those would be currently outside scope of this article. This article is specifically targeting Paterson’s worm, which is one very particular type of SAW. For that reason, it can clearly be seen that we have in this work produced more than enough content (10 original figures) just on Paterson’s worm alone, and this article is only one in our Paterson’s worm research, so covering the whole background of SAW and adding more references for SAW could be related and interesting, but to increase the focus, we chose not to include these background introduction material as we already have more than enough scope and results for this original article. For adding more background, we may need to remove some of our own work and original results.

Due to lack of quantitative justification of the results, the motivation of the study performed and lack of generalization, I cannot recommend this paper for publication. My advice to the author will be to investigate the SAW and to place adequately his work within the field, as well as add statistical methods and valid arguments of the scientific soundness of his research into the text.

Several advantages of the work remain, including the new grid, original results and implementations of the proposed system for future researchers to explore.
