# Supplementary material for: Mathematical model for worm path simulation in 3D
Source: PLoS One. 2026 Sep 11;21(9):e0358065. doi: 10.1371/journal.pone.0358065 (PMC13568494; doi:10.1371/journal.pone.0358065)
Supplement: Peer review history

# Author comment: Author response to Decision Letter 0

**DOI:** 10.1371/journal.pone.0358065.r001

**Transfer Alert**

This paper was transferred from another journal. As a result, its full editorial history (including decision letters, peer reviews and author responses) may not be present.

13 Mar 2026

# Peer review: Decision Letter 0

**DOI:** 10.1371/journal.pone.0358065.r002

**Authors:** Rui Lu

17 May 2026

Dear Dr. Vaughan,

Please submit your revised manuscript by Jul 01 2026 11:59PM. If you will need more time than this to complete your revisions, please reply to this message or contact the journal office at plosone@plos.org. When you're ready to submit your revision, log on to https://www.editorialmanager.com/pone/ and select the 'Submissions Needing Revision' folder to locate your manuscript file.

- A letter that responds to each point raised by the academic editor and reviewer(s). You should upload this letter as a separate file labeled 'Response to Reviewers'.
- A marked-up copy of your manuscript that highlights changes made to the original version. You should upload this as a separate file labeled 'Revised Manuscript with Track Changes'.
- An unmarked version of your revised paper without tracked changes. You should upload this as a separate file labeled 'Manuscript'.

If you would like to make changes to your financial disclosure, please include your updated statement in your cover letter. Guidelines for resubmitting your figure files are available below the reviewer comments at the end of this letter.

If applicable, we recommend that you deposit your laboratory protocols in protocols.io to enhance the reproducibility of your results. Protocols.io assigns your protocol its own identifier (DOI) so that it can be cited independently in the future. For instructions see: https://journals.plos.org/plosone/s/submission-guidelines#loc-laboratory-protocols. Additionally, PLOS ONE offers an option for publishing peer-reviewed Lab Protocol articles, which describe protocols hosted on protocols.io. Read more information on sharing protocols at https://plos.org/protocols?utm_medium=editorial-email&utm_source=authorletters&utm_campaign=protocols.

As the corresponding author, your ORCID iD is verified in the submission system and will appear in the published article. PLOS supports the use of ORCID, and we encourage all coauthors to register for an ORCID iD and use it as well. Please encourage your coauthors to verify their ORCID iD within the submission system before final acceptance, as unverified ORCID iDs will not appear in the published article. *Only*  the individual author can complete the verification step; PLOS staff *cannot*  verify ORCID iDs on behalf of authors.

We look forward to receiving your revised manuscript.

Kind regards,

Rui Lu

Academic Editor

PLOS One

Journal Requirements:

When submitting your revision, we need you to address these additional requirements.

1. Please ensure that your manuscript meets PLOS ONE's style requirements, including those for file naming. The PLOS ONE style templates can be found at

https://journals.plos.org/plosone/s/file?id=wjVg/PLOSOne_formatting_sample_main_body.pdf and https://journals.plos.org/plosone/s/file?id=ba62/PLOSOne_formatting_sample_title_authors_affiliations.pdf

2. In the online submission form, you indicated that the data presented in this study is available on request from the corresponding author. The data is not publicly available due to the privacy restrictions of the collaborating organization and safety regulations concerning infrastructure data.

All PLOS journals now require all data underlying the findings described in their manuscript to be freely available to other researchers, either 1. In a public repository, 2. Within the manuscript itself, or 3. Uploaded as supplementary information.

This policy applies to all data except where public deposition would breach compliance with the protocol approved by your research ethics board. If your data cannot be made publicly available for ethical or legal reasons (e.g., public availability would compromise patient privacy), please explain your reasons on resubmission and your exemption request will be escalated for approval.

3. Thank you for stating the following financial disclosure:

This research was funded by the Science and Technology Program of Guangxi, China (No. 2023AB08158)

Please state what role the funders took in the study. If the funders had no role, please state: "The funders had no role in study design, data collection and analysis, decision to publish, or preparation of the manuscript."

If this statement is not correct you must amend it as needed.

Please include this amended Role of Funder statement in your cover letter; we will change the online submission form on your behalf.

4. We note that Figure(s) 1, 2, 3, 4, 7 in your submission contain copyrighted images. All PLOS content is published under the Creative Commons Attribution License (CC BY 4.0), which means that the manuscript, images, and Supporting Information files will be freely available online, and any third party is permitted to access, download, copy, distribute, and use these materials in any way, even commercially, with proper attribution. For more information, see our copyright guidelines: http://journals.plos.org/plosone/s/licenses-and-copyright.

We require you to either (1) present written permission from the copyright holder to publish these figures specifically under the CC BY 4.0 license, or (2) remove the figures from your submission:

a. You may seek permission from the original copyright holder of Figure(s) 1, 2, 3, 4, 7 to publish the content specifically under the CC BY 4.0 license.

We recommend that you contact the original copyright holder with the Content Permission Form (http://journals.plos.org/plosone/s/file?id=7c09/content-permission-form.pdf) and the following text:

“I request permission for the open-access journal PLOS ONE to publish XXX under the Creative Commons Attribution License (CCAL) CC BY 4.0 (http://creativecommons.org/licenses/by/4.0/). Please be aware that this license allows unrestricted use and distribution, even commercially, by third parties. Please reply and provide explicit written permission to publish XXX under a CC BY license and complete the attached form.”

Please upload the completed Content Permission Form or other proof of granted permissions as an "Other" file with your submission.

In the figure caption of the copyrighted figure, please include the following text: “Reprinted from [ref] under a CC BY license, with permission from [name of publisher], original copyright [original copyright year].”

b. If you are unable to obtain permission from the original copyright holder to publish these figures under the CC BY 4.0 license or if the copyright holder’s requirements are incompatible with the CC BY 4.0 license, please either i) remove the figure or ii) supply a replacement figure that complies with the CC BY 4.0 license. Please check copyright information on all replacement figures and update the figure caption with source information. If applicable, please specify in the figure caption text when a figure is similar but not identical to the original image and is therefore for illustrative purposes only.

5. Please note that PLOS One has specific guidelines on code sharing for submissions in which author-generated code underpins the findings in the manuscript. In these cases, we expect all author-generated code to be made available without restrictions upon publication of the work. Please review our guidelines at https://journals.plos.org/plosone/s/materials-and-software-sharing#loc-sharing-code and ensure that your code is shared in a way that follows best practice and facilitates reproducibility and reuse.

6. Please provide a complete Data Availability Statement in the submission form, ensuring you include all necessary access information or a reason for why you are unable to make your data freely accessible. If your research concerns only data provided within your submission, please write "All data are in the manuscript and/or supporting information files" as your Data Availability Statement.

7. Thank you for stating the following in the Acknowledgments Section of your manuscript:

This research was supported by the Royal Academy of Engineering (RAEng) through a research fellowship awarded to N. V.

We note that you have provided funding information that is not currently declared in your Funding Statement. However, funding information should not appear in the Acknowledgments section or other areas of your manuscript. We will only publish funding information present in the Funding Statement section of the online submission form.

Please remove any funding-related text from the manuscript and let us know how you would like to update your Funding Statement. Currently, your Funding Statement reads as follows:

Royal Academy of Engineering (RAEng) Research Fellowship to NV

Please include your amended statements within your cover letter; we will change the online submission form on your behalf.

8. Please update your submission to use the PLOS LaTeX template. The template and more information on our requirements for LaTeX submissions can be found at http://journals.plos.org/plosone/s/latex.

9. We note that Figure(s) 1-9 in your submission contain copyrighted images. All PLOS content is published under the Creative Commons Attribution License (CC BY 4.0), which means that the manuscript, images, and Supporting Information files will be freely available online, and any third party is permitted to access, download, copy, distribute, and use these materials in any way, even commercially, with proper attribution. For more information, see our copyright guidelines: http://journals.plos.org/plosone/s/licenses-and-copyright.

We require you to either (1) present written permission from the copyright holder to publish these figures specifically under the CC BY 4.0 license, or (2) remove the figures from your submission:

a. You may seek permission from the original copyright holder of Figure(s) 1-9 to publish the content specifically under the CC BY 4.0 license.

We recommend that you contact the original copyright holder with the Content Permission Form (http://journals.plos.org/plosone/s/file?id=7c09/content-permission-form.pdf) and the following text:

“I request permission for the open-access journal PLOS ONE to publish XXX under the Creative Commons Attribution License (CCAL) CC BY 4.0 (http://creativecommons.org/licenses/by/4.0/). Please be aware that this license allows unrestricted use and distribution, even commercially, by third parties. Please reply and provide explicit written permission to publish XXX under a CC BY license and complete the attached form.”

Please upload the completed Content Permission Form or other proof of granted permissions as an "Other" file with your submission.

In the figure caption of the copyrighted figure, please include the following text: “Reprinted from [ref] under a CC BY license, with permission from [name of publisher], original copyright [original copyright year].”

b. If you are unable to obtain permission from the original copyright holder to publish these figures under the CC BY 4.0 license or if the copyright holder’s requirements are incompatible with the CC BY 4.0 license, please either i) remove the figure or ii) supply a replacement figure that complies with the CC BY 4.0 license. Please check copyright information on all replacement figures and update the figure caption with source information. If applicable, please specify in the figure caption text when a figure is similar but not identical to the original image and is therefore for illustrative purposes only.

10. Please include captions for your Supporting Information files at the end of your manuscript, and update any in-text citations to match accordingly. Please see our Supporting Information guidelines for more information: http://journals.plos.org/plosone/s/supporting-information.

11. If the reviewer comments include a recommendation to cite specific previously published works, please review and evaluate these publications to determine whether they are relevant and should be cited. There is no requirement to cite these works unless the editor has indicated otherwise.

[Note: HTML markup is below. Please do not edit.]

Reviewers' comments:

Reviewer's Responses to Questions

**Comments to the Author**

1. Is the manuscript technically sound, and do the data support the conclusions?

Reviewer #1: Yes

Reviewer #2: Partly

Reviewer #3: No

**********

2. Has the statistical analysis been performed appropriately and rigorously?

Reviewer #1: N/A

Reviewer #2: No

Reviewer #3: No

**********

3. Have the authors made all data underlying the findings in their manuscript fully available?

The PLOS Data policy

Reviewer #1: No

Reviewer #2: Yes

Reviewer #3: Yes

**********

4. Is the manuscript presented in an intelligible fashion and written in standard English?

Reviewer #1: Yes

Reviewer #2: Yes

Reviewer #3: Yes

**********

Reviewer #1: Re #3: The author did not provide all code used in this work. Furthermore, the code is not publicly available, though I do not consider that an issue at the review stage.

Please see my attached comments for further info regarding the Major Revision decision.

Reviewer #2: This paper extends the classical Paterson worm model from 2D grids to a 3D cubic orthogonal lattice. Through exhaustive recursive simulation, the author reports 3239 unique worms and classifies their outcomes into terminating, looping, and running categories. However, several concerns limit the strength of the contribution. The reliability of the reported results is not independently verified. Some theoretical claims, such as the 13-rule upper bound, are not supported by rigorous mathematical proof. The biological motivation and application for extending the model to 3D requires further clarification.

1. The biological motivation for extending the worm foraging problem to 3D should be explained more clearly. It is unclear what real-world biological or physical process corresponds to discrete orthogonal jumps on a 3D cubic lattice. How can a worm move through 3D space without any supporting medium or substrate? Grounding the model in a concrete real-world scenario would strengthen the motivation for this research.

2. All results in this paper are based on a single fixed set of hyperparameters. For example: the population limit (53.5M steps), the loop detection thresholds (≥4 repetitions, ≥3 rules per cycle), and the manually identified mirror symmetries. A sensitivity analysis showing how the reported numbers change under different parameter choices would be important for assessing the robustness of the conclusions.

3. The purpose of the random search experiment at population limit 2,100 is unclear. Can the resulting T/L/R proportions be compared against a recursive search at the same population limit? Such a direct comparison would help clarify what this experiment contributes beyond the recursive results.

4. In Section 1.4, the author claims "It is shown with a proof that no worms in this 3D grid can use more than 13 rules." However, the argument that follows is an informal verbal explanation rather than a rigorous mathematical proof. A formal proof structure is needed to support this claim.

5. The quality of all figures in the paper is insufficient. The individual worm structures are difficult to distinguish. The figures should be reworked at higher resolution and clarity to convey the intended information.

Reviewer #3: The manuscript presents a computational study on a 3D orthogonal grid, framed as simulations of worm paths. The author extends previous 2D triangular/hexagonal grid models to 3D, enumerating 3239 unique worm configurations and classifying their outcomes.

The problem of this work which cannot be easily fixed is its narrow scope. All the experiments performed are held in very particular conditions (e.g., a reader can see a lot of arbitrary numbers as parameter values which are not justified). The author does not place his work into a broader scientific scope and fails to generalize his results. In fact, the author's work falls into the scientific area of self-avoiding walks (SAW) which is not mentioned anyhow. Based on the fact that the references list is rather short (14 papers), I assume that the author was not thorough enough when analyzing the corresponding works of other researchers.

Due to lack of quantitative justification of the results, the motivation of the study performed and lack of generalization, I cannot recommend this paper for publication. My advice to the author will be to investigate the SAW and to place adequately his work within the field, as well as add statistical methods and valid arguments of the scientific soundness of his research into the text.

**********

what does this mean?). If published, this will include your full peer review and any attached files.

If you choose “no”, your identity will remain anonymous but your review may still be made public.

**Do you want your identity to be public for this peer review?**  For information about this choice, including consent withdrawal, please see our Privacy Policy

Reviewer #1: No

Reviewer #2: No

Reviewer #3: No

**********

[NOTE: If reviewer comments were submitted as an attachment file, they will be attached to this email and accessible via the submission site. Please log into your account, locate the manuscript record, and check for the action link "View Attachments". If this link does not appear, there are no attachment files.]

To ensure your figures meet our technical requirements, please review our figure guidelines: https://journals.plos.org/plosone/s/figures

You may also use PLOS’s free figure tool, NAAS, to help you prepare publication quality figures: https://journals.plos.org/plosone/s/figures#loc-tools-for-figure-preparation.

NAAS will assess whether your figures meet our technical requirements by comparing each figure against our figure specifications.

Attachment Submitted filename: WormsReview.docx

# Author comment: Author response to Decision Letter 1

**DOI:** 10.1371/journal.pone.0358065.r003

13 Jul 2026

The source code repository now has a permanent DOI and is included in the manuscript as reference [11]:

[11] Vaughan N, (2026-07-13), Mathematical model for Worm Path Simulation in 3D, Zenodo Repository of JavaScript C Source Code and Android APK, https://doi.org/10.5281/zenodo.21335902

Below is the detailed response to reviewers.

Also this is attached as Rebuttal.docx.

WormsReview.doc

The research presented here looks at possible 3D worm paths on an orthogonal grid. Previous work has focused exclusively on 2D grids with various tilings. Such a grid better reflects the spatial complexity of the space that worms occupy and is thus of importance for understanding their movement. The author explores how a deterministic, rule-based algorithm with <= 32 parameters controls the worm movements. Worms are classified as Terminated, Looping, or Running depending on the outcome of the simulation. The authors present interesting worms and set the stage for future work to explore the types of worms in this model. Additionally, virtual reality and 3D visualization is created to allow for immersive exploration of the paths. The JavaScript is helpful for understanding some of the algorithm.

Thank you for this summary description

The paper lacks technical descriptions of many aspects, and those that are provided do not offer sufficient clarity. Examples include “chaotically” and “population”.

I have now added definitions of both chaotically and population in multiple places in the manuscript.

After reviewing, I still do not understand what a “rule” is. It would seem to me that for the worm that immediately completes a square to return to the origin, that when it returns to the origin, it must select a new rule that continues it on its path since 4 such paths are available at that time. That is how I read “The worm continues in this way and if a situation is encountered where no paths are available, the worm terminates.”

Yes this is correct. An example of a complete rule is something like : 10403030151. Each number represents which path (from 0 to 5) should be chosen at each decision point. The first path would be path 1, then when another choice is needed, path 0 would be chosen as that is the next number. I have now modified the text to clarify this.

Another key limitation of the presented work is lack of figures that summarize the analyses that are suggested in the text. At minimum, summaries of how many worms end up in each category should be shown. Given how central the idea of “population” and/or “time” is, showing how this evolves over time would be useful. Finally, loop length.

I agree this would help to visualise and clarify. Added to manuscript: “Fig. 10. Stacked area chart showing that when worms are run for a longer time (to a larger population with a larger number of lines per worm), the proportion of worms in each category changes, and finally, 58% of all worms end as category T (terminated), 42% of worms end as category L (looping repetition) and only about 0.1% of worms (5 individual worms) remain in category R (still running with unknown outcome) beyond 53.5 million which was as far as the current hardware could explore.”

Overall, this paper has much to improve upon to make it a stronger contribution to the field and to inform readers.

1. The exposition of various aspects of the model and its analysis are provided in a convoluted ordering, making it difficult to comprehend for a reader new to the field.

2. The worm codes are not explained. Is there a special meaning to “14305144543” for example that can be parsed by readers? If not, summaries across the various grouping of worms would provide more informative visualizations. For example, rather than Table 1, a histogram of Loop Size over all category L worms would be more informative.

Yes the numbers such as 10403030151 are examples of rules which produces a specific worm path. Each number represents which path (from 0 to 5) should be chosen at each decision point. The first path would be path 1, then when another choice is needed, path 0 would be chosen as that is the next number. Recording these makes the worms reproducible and enables counting the unique worms.

3. This also raises another concern: all the figures are various renderings of the paths that worms take. There are no variations that provide a greater context for these results as the author has done in previous publications.

4. “whenever the same situation is encountered.” I have an intuition about what this means for the worm’s rules, but it would help to state this more explicitly. I interpret it to mean “whenever the same set of available paths is encountered.”

I agree, manuscript updated to replace ‘situation’ with ‘set of available paths.’

5. It might be beyond the author’s control as the field may have already set this, but the 0-5 “paths” and the numbered edges of the worm’s overall path feels semantically overloaded.

6. The concept of “population” is not defined. For someone outside this field, this is an unintuitive use of this word.

Added to section 1.1: “Population means the number of lines in the worm.”

7. Is there a technical definition for “chaos” in this field? Providing one here would help readers understand the precise point the author is making.

Added a definition of ‘chaotic’: “By chaotic, we mean moving without any foreseeable pattern and no repetition, such that the outcome cannot be known without completing the simulation.”

8. The language of the abstract and conclusion is too informal for a research paper. To give specifics: 1) Use of contractions. 2) Describing worms as “interesting” rather than a more technical term such as “unique” or “outlier”.

Removed the contractions including replaced “haven’t” with “have not.” Replaced ‘interesting’ with ‘unique outlier.’

9. I believe a 3D orthogonal grid with diagonals allowed will either provide 26 or—if <1,1,1>-type diagonals are excluded—18 paths are allowed. That’s what my Rubik’s cube says.

This may be correct and interesting for future research including diagonals on a cube. That number 26 would also need to be reduced since retracing the path is not allowed, so it may have a maximum of 25 available path choices. Currently in this 3D cube research, diagonals were not allowed. We have previously explored diagonals on a 2D grid, and would be interesting to look into that in 3D. Even with 6 or 8 paths the number of possible worms is huge, so with 26 paths, it would only be possible to explore a microscopic proportion of the astronomical number of potential worms.

3. Thank you for stating the following financial disclosure:

This research was funded by the Science and Technology Program of Guangxi, China (No. 2023AB08158)

This is not correct. In the manuscript, the acknowledgement states this: “The research was supported by the Royal Academy of Engineering (RAEng) through a research fellowship awarded to N. V.”

Please state what role the funders took in the study. If the funders had no role, please state: "The funders had no role in study design, data collection and analysis, decision to publish, or preparation of the manuscript."

If this statement is not correct you must amend it as needed.

Please include this amended Role of Funder statement in your cover letter; we will change the online submission form on your behalf.

I have added this into the manuscript under acknowledgements: “The funders had no role in study design, data collection and analysis, decision to publish, or preparation of the manuscript.”

4. We note that Figure(s) 1, 2, 3, 4, 7 in your submission contain copyrighted images. All PLOS content is published under the Creative Commons Attribution License (CC BY 4.0).

This does not apply – All figures in this manuscript are original and unpublished, custom-made for this publication only.

5. Please note that PLOS One has specific guidelines on code sharing for submissions in which author-generated code underpins the findings in the manuscript. In these cases, we expect all author-generated code to be made available without restrictions upon publication of the work. Please review our guidelines at https://journals.plos.org/plosone/s/materials-and-software-sharing#loc-sharing-code and ensure that your code is shared in a way that follows best practice and facilitates reproducibility and reuse.

As requested, the author has now shared all the code in public domain accessible through reference [12], including .APK android file, JavaScript, and C code, all unpublished and custom made for this publication only.

6. Please provide a complete Data Availability Statement in the submission form, ensuring you include all necessary access information or a reason for why you are unable to make your data freely accessible. If your research concerns only data provided within your submission, please write "All data are in the manuscript and/or supporting information files" as your Data Availability Statement.

The author has shared all the code in public domain accessible through reference [12].

7. Thank you for stating the following in the Acknowledgments Section of your manuscript:

This research was supported by the Royal Academy of Engineering (RAEng) through a research fellowship awarded to N. V.

We note that you have provided funding information that is not currently declared in your Funding Statement. However, funding information should not appear in the Acknowledgments section or other areas of your manuscript. We will only publish funding information present in the Funding Statement section of the online submission form.

Please remove any funding-related text from the manuscript and let us know how you would like to update your Funding Statement. Currently, your Funding Statement reads as follows:

Royal Academy of Engineering (RAEng) Research Fellowship to NV

Please include your amended statements within your cover letter; we will change the online submission form on your behalf.

Please include this funding acknowledgement:

“This research was supported by the Royal Academy of Engineering (RAEng) through a research fellowship awarded to N. V. The funders had no role in study design, data collection and analysis, decision to publish, or preparation of the manuscript.”

8. Please update your submission to use the PLOS LaTeX template. The template and more information on our requirements for LaTeX submissions can be found at http://journals.plos.org/plosone/s/latex.

9. We note that Figure(s) 1-9 in your submission contain copyrighted images. All PLOS content is published under the Creative Commons Attribution License (CC BY 4.0), which means that the manuscript, images, and Supporting Information files will be freely available online, and any third party is permitted to access, download, copy, distribute, and use these materials in any way, even commercially, with proper attribution. For more information, see our copyright guidelines: http://journals.plos.org/plosone/s/licenses-and-copyright.

N/A all figures are original and unpublished.

10. Please include captions for your Supporting Information files at the end of your manuscript, and update any in-text citations to match accordingly. Please see our Supporting Information guidelines for more information: http://journals.plos.org/plosone/s/supporting-information.

11. If the reviewer comments include a recommendation to cite specific previously published works, please review and evaluate these publications to determine whether they are relevant and should be cited. There is no requirement to cite these works unless the editor has indicated otherwise.

5. Review Comments to the Author

Reviewer #1: Re #3: The author did not provide all code used in this work. Furthermore, the code is not publicly available, though I do not consider that an issue at the review stage.

As requested, the author has now shared all the code in public domain accessible through reference [12], including .APK android file, javascript, and C code, all unpublished and custom made for this publication only.

Please see my attached comments for further info regarding the Major Revision decision.

Reviewer #2: This paper extends the classical Paterson worm model from 2D grids to a 3D cubic orthogonal lattice. Through exhaustive recursive simulation, the author reports 3239 unique worms and classifies their outcomes into terminating, looping, and running categories. However, several concerns limit the strength of the contribution. The reliability of the reported results is not independently verified.

This is an original work, and no other researchers have worked on this topic, so there is no independent verification. However the code is fully shared which fully enables any future researchers to replicate and independently verify or build on this further.

Some theoretical claims, such as the 13-rule upper bound, are not supported by rigorous mathematical proof.

I agree, in response, we have now removed “with a proof” as this is not an official proof.

A description of the proof is given by the author very clearly and precisely and this is strong enough to conclusively prove that 13 is the maximum possible rule upper bound, without any doubt, this is an original new finding (included in manuscript):

It is shown with a proof that no worms in this 3D grid can use more than 13 rules. Worms have 6 binary sensors but path 2 is always unavailable, which leaves 25 possible rule choices. In theory up to 32 binary rules are available, because there are maximum of 32 possible binary rule representations from 00000 to 11111. However, half of these rules have an even number of paths available, so only 2 of these can be used per worm, because an even number of available paths can only occur when passing through the origin, which cannot happen more than twice. Of the remaining 16 rules with an odd number of paths, 5 of those rules have only 1 path which are automatically selected without rule setting. One rule has 5 paths which is the first rule, it is restricted to 1 and it does count as a rule. Additionally, this leaves 10 rules with 3 paths. In addition a worm could use 2 rules with an even number of paths: 4 and 2 which can occur.

This shows that the total maximum number of rules that could be used by one worm is restricted to 13.

The biological motivation and application for extending the model to 3D requires further clarification.

This is certainly a bio-inspired model but sits at the interface of biology and mathematics. Like the original Paterson’s worm in 2D, there are some differences between this abstract mathematical model and the actual biological workings in-vivo real worms. However the main take-aways from proposing and analysing a model such as this, are still profound and meaningful, as stated in the manuscript, that this work clarifies that “Simple rules result in surprisingly complex and intricate movements.” And “Images and interesting observations are presented demonstrating complex 3D patterns that emerge.”

1. The biological motivation for extending the worm foraging problem to 3D should be explained more clearly. It is unclear what real-world biological or physical process corresponds to discrete orthogonal jumps on a 3D cubic lattice. How can a worm move through 3D space without any supporting medium or substrate? Grounding the model in a concrete real-world scenario would strengthen the motivation for this research.

Worms are not expected to “move through 3D space without any supporting medium or substrate.” In real worms, they burrow underground, worms can clearly burrow tunnels horizontally as well as vertically, so in-vivo worms exist in a 3D soil substrate. From this perspective the original 2D Paterson’s worms seems very restrictive as only surface dwelling creatures are restricted to 2D movement, worms which exist within the underground soil are moving in 3D.

2. All results in this paper are based on a single fixed set of hyperparameters. For example: the population limit (53.5M steps), the loop detection thresholds (≥4 repetitions, ≥3 rules per cycle), and the manually identified mirror symmetries. A sensitivity analysis showing how the reported numbers change under different parameter choices would be important for assessing th

Attachment Submitted filename: Rebuttal.docx

# Peer review: Decision Letter 1

**DOI:** 10.1371/journal.pone.0358065.r004

**Authors:** Rui Lu

26 Aug 2026

Mathematical model for Worm Path Simulation in 3D

PONE-D-26-12101R1

Dear Dr. Neil Vaughan,

We’re pleased to inform you that your manuscript has been judged scientifically suitable for publication and will be formally accepted for publication once it meets all outstanding technical requirements.

Within one week, you’ll receive an e-mail detailing the required amendments. When these have been addressed, you’ll receive a formal acceptance letter and your manuscript will be scheduled for publication.

An invoice will be generated when your article is formally accepted. Please note, if your institution has a publishing partnership with PLOS and your article meets the relevant criteria, all or part of your publication costs will be covered. Please make sure your user information is up-to-date by logging into Editorial Manager at Editorial Manager® and clicking the ‘Update My Information' link at the top of the page. For questions related to billing, please contact billing support.

If your institution or institutions have a press office, please notify them about your upcoming paper to help maximize its impact. If they’ll be preparing press materials, please inform our press team as soon as possible -- no later than 48 hours after receiving the formal acceptance. Your manuscript will remain under strict press embargo until 2 pm Eastern Time on the date of publication. For more information, please contact onepress@plos.org.

Kind regards,

Rui Lu

Academic Editor

PLOS One

Additional Editor Comments (optional):

Reviewers' comments:

Reviewer's Responses to Questions

**Comments to the Author**

Reviewer #1: All comments have been addressed

Reviewer #2: All comments have been addressed

Reviewer #3: (No Response)

**********

2. Is the manuscript technically sound, and do the data support the conclusions?

Reviewer #1: Yes

Reviewer #2: Yes

Reviewer #3: No

**********

3. Has the statistical analysis been performed appropriately and rigorously?

Reviewer #1: Yes

Reviewer #2: Yes

Reviewer #3: No

**********

4. Have the authors made all data underlying the findings in their manuscript fully available?

The PLOS Data policy

Reviewer #1: Yes

Reviewer #2: Yes

Reviewer #3: Yes

**********

5. Is the manuscript presented in an intelligible fashion and written in standard English?

Reviewer #1: Yes

Reviewer #2: Yes

Reviewer #3: Yes

**********

Reviewer #1: The author has addressed my previous comments in a satisfactory manner. There is now greater clarity for readers unfamiliar with this area of research. I do still think the word codes could be explained in a way that makes their numeric representation more meaningful to readers such as myself who are not embedded in this field. That said, arriving at a first-order understanding of them is within reach of all readers. Hence, I do not consider this as disqualifying for publication.

I do really appreciate the new Figure 10. I think it adds really useful information about how these analyses evolve as the population is allowed to grow. I do think more figures like this in the future will better push the field forward. For example, proportion of worms in R as population --> inf on a log-scale.

One minor comment: there is a typo on the y-label on Figure 10. "Propostion" should be "Proportion".

Reviewer #2: (No Response)

Reviewer #3: The authors did make some cosmetic changes to the revised version of the text, however, the most worrisome issues I've mentioned in the previous review are not properly addressed. Therefore, I have to retain my previous verdict.

**********

what does this mean?). If published, this will include your full peer review and any attached files.

If you choose “no”, your identity will remain anonymous but your review may still be made public.

**Do you want your identity to be public for this peer review?**  For information about this choice, including consent withdrawal, please see our Privacy Policy

Reviewer #1: No

Reviewer #2: No

Reviewer #3: No

**********

# Editor report: Acceptance letter

**DOI:** 10.1371/journal.pone.0358065.r005

**Authors:** Rui Lu

PONE-D-26-12101R1

PLOS One

Dear Dr. Vaughan,

I'm pleased to inform you that your manuscript has been deemed suitable for publication in PLOS One. Congratulations! Your manuscript is now being handed over to our production team.

At this stage, our production department will prepare your paper for publication. This includes ensuring the following:

* All references, tables, and figures are properly cited

* All relevant supporting information is included in the manuscript submission,

* There are no issues that prevent the paper from being properly typeset

You will receive further instructions from the production team, including instructions on how to review your proof when it is ready. Please keep in mind that we are working through a large volume of accepted articles, so please give us a few days to review your paper and let you know the next and final steps.

Lastly, if your institution or institutions have a press office, please let them know about your upcoming paper now to help maximize its impact. If they'll be preparing press materials, please inform our press team within the next 48 hours. Your manuscript will remain under strict press embargo until 2 pm Eastern Time on the date of publication. For more information, please contact onepress@plos.org.

You will receive an invoice from PLOS for your publication fee after your manuscript has reached the completed accept phase. If you receive an email requesting payment before acceptance or for any other service, this may be a phishing scheme. Learn how to identify phishing emails and protect your accounts at https://explore.plos.org/phishing.

If we can help with anything else, please email us at customercare@plos.org.

Thank you for submitting your work to PLOS One and supporting open access.

Kind regards,

PLOS One Editorial Office Staff

on behalf of

Dr. Rui Lu

Academic Editor

PLOS One
